# Comparative transcriptomics reveals key differences in the response to milk oligosaccharides of infant gut-associated bifidobacteria

**DOI:** 10.1038/srep13517

**Published:** 2015-09-04

**Authors:** Daniel Garrido, Santiago Ruiz-Moyano, Danielle G. Lemay, David A. Sela, J. Bruce German, David A. Mills

**Affiliations:** 1 Department of Viticulture & Enology, One Shields Ave. Davis, CA 95616, United States; 2 Food Science & Technology, One Shields Ave. Davis, CA 95616, United States; 3 Foods for Health Institute, One Shields Ave. Davis, CA 95616, United States; 4 Genome Center, University of California, One Shields Ave. Davis, CA 95616, United States; 5 Department of Chemical and Bioprocess Engineering, School of Engineering, Av. Vicuña Mackenna 4860, Santiago, Chile; 6 Programa ASIS, Pontificia Universidad Católica de Chile, Av. Vicuña Mackenna 4860, Santiago, Chile; 7 Department of Food Science, University of Massachusetts, Amherst, MA 01003.

## Abstract

Breast milk enhances the predominance of *Bifidobacterium* species in the infant gut, probably due to its large concentration of human milk oligosaccharides (HMO). Here we screened infant-gut isolates of *Bifidobacterium longum* subsp. *infantis* and *Bifidobacterium bifidum* using individual HMO, and compared the global transcriptomes of representative isolates on major HMO by RNA-seq. While *B. infantis* displayed homogeneous HMO-utilization patterns, *B. bifidum* were more diverse and some strains did not use fucosyllactose (FL) or sialyllactose (SL). Transcriptomes of *B. bifidum* SC555 and *B. infantis* ATCC 15697 showed that utilization of pooled HMO is similar to neutral HMO, while transcriptomes for growth on FL were more similar to lactose than HMO in *B. bifidum*. Genes linked to HMO-utilization were upregulated by neutral HMO and SL, but not by FL in both species. In contrast, FL induced the expression of alternative gene clusters in *B. infantis*. Results also suggest that *B. bifidum* SC555 does not utilize fucose or sialic acid from HMO. Surprisingly, expression of orthologous genes differed between both bifidobacteria even when grown on identical substrates. This study highlights two major strategies found in *Bifidobacterium* species to process HMO, and presents detailed information on the close relationship between HMO and infant-gut bifidobacteria.

Breast milk is a complex fluid, shaped by evolution to meet offspring needs regarding nutrition and immune protection. Human milk consists of a myriad of molecules with varying structure and abundance, present in a very concentrated form^1^. Several of these compounds are considered to be bioactive agents, potentially playing important roles beyond nutrition by promoting immunological protection and stimulating proper development^2^,^3^.

Free human milk oligosaccharides (HMO) are among the most abundant components in human milk^4^, after water and lactose. These are carbohydrates with a degree of polymerization from 3 to 32, composed of five monomers: D-glucose (Glc), D-galactose (Gal), *N-*acetylglucosamine (GlcNAc), L-fucose (Fuc) and *N-*acetylneuraminic acid (Neu5Ac, or sialic acid). The combinatory potential of structural isomers is high, and HMO represent a large catalogue of complex carbohydrates. However, no more than 50 HMO species are normally abundant in an individual mother’s milk^5^. Major HMO secreted in milk include lacto-*N-*tetraose (LNT), lacto-*N*-neotetraose (LNnT) and lacto-*N-*hexaose, which are neutral HMO, in addition to fucosylated molecules such as 2-fucosyllactose (2FL), 3-fucosyllactose (3FL), and lacto-*N-*fucopentaoses I, II and III^5^. Finally, sialyl-lacto-*N-*tetraose, 3′ and 6′sialyllactose (6SL) are examples of acidic HMO^6^.

Interestingly, the energetic value derived from conventional digestion of these molecules is negligible for the nursing infant. Intestinal enzymes are incapable of breaking down the majority of complex linkages found in HMO^7^. While initially puzzling for scientists, several major functions have emerged and attributed to these molecules. For example, HMO can prevent pathogen binding to the intestinal epithelium given their structural similarity with glycoconjugates in the brush border^2^.

In addition, HMO are substrates for beneficial microbes in the developing infant colon^8^. Studies have consistently shown that the composition of the intestinal microbiome among breast-fed and formula-fed infants is different^9^,^10^,^11^. This is consistent with HMO enriching for the populations capable of efficiently utilizing these substrates.

In general, it is understood that species of the *Bifidobacterium* genus dominate the infant gut microbiota in the first year of life^12^,^13^,^14^. Interestingly, there are only a few bifidobacterial species that are consistently isolated from infant feces, including *Bifidobacterium longum* subsp. *longum* (*B. longum*), *Bifidobacterium longum* subsp. *infantis* (*B. infantis*), *Bifidobacterium breve*, and *Bifidobacterium bifidum,* among other lesser taxa^11^,^15^,^16^,^17^.

It has been previously shown that certain infant-associated bifidobacteria can utilize HMO purified from breast milk as the sole carbon source^18^. This first suggested potential co-evolution between bifidobacteria and their mammalian hosts mediated by milk oligosaccharides. This consumption phenotype was observed for some *B. infantis* and *B. bifidum* strains^19^,^20^, and recently it has been shown that also several *B. breve* can target these molecules, including fucosylated HMO^21^.

Comparative genomics and functional studies have been very useful in providing a molecular framework underlying bifidobacterial HMO utilization, particularly for *B. infantis* ATCC 15697^22^ and *B. bifidum* PRL2010^20^. *B. infantis* deploys a HMO utilization strategy involving translocation of intact oligosaccharides using ABC transporters^23^. Once internalized, milk oligosaccharides are degraded by several intracellular glycosyl hydrolases that target linkages inherent to HMO^24^,^25^. Released monosaccharides are fermented in the central fructose-6-phosphate phosphoketolase pathway also referred to as the “bifid shunt”, resulting in the production of ATP and secretion of acetate and lactate as end products. In contrast, the HMO consumption strategy of *B. bifidum* differs dramatically from *B. infantis*^24^. In *B. bifidum*, HMO are initially processed extracellularly, primarily via secreted glycosyl hydrolases that degrade HMO into constituent mono- and disaccharides. *B. bifidum* then imports the resultant products to be catabolized intracellularly through the bifid shunt^26^.

In order to gain a fundamental understanding of how these two different bifidobacteria have adapted to milk oligosaccharide utilization and therefore grow in the infant intestinal environment, it is essential to address the diversity of phenotypes found in infant-gut associated strains and also to dissect the global transcriptional responses of these microorganisms to individual and chemically different oligosaccharides found in breast milk. In this study, we determined the ability of *B. bifidum* and *B. infantis* isolates to utilize pooled HMO and the synthetic HMO LNT, LNnT, 2FL, 3FL and 6SL as well as mucin. In addition, we detailed genome-wide transcriptional responses of *B. bifidum* and *B. infantis* during growth on these HMO substrates as the sole carbon source.

## Results

### Growth of *B. infantis* and *B. bifidum* on pooled and individual HMO

In a previous study, we created a collection of 461 *Bifidobacterium* isolates from 40 breast-fed infant fecal samples^21^. Among these, 297 were identified as *B. longum* (subsp. *infantis* or subsp. *longum*), and 22 as *B. bifidum*^21^. In order to discriminate these isolates at the strain level, Multilocus Sequencing Typing (MLST) analysis^27^ was performed on purified genomic DNA of these isolates as well as 15 other strains obtained from culture collections (Table S1). Isolates from unrelated infants that shared identical MLST profiles were conservatively considered as separate isolates in this study. The MLST analysis identified 5 *B. bifidum* and 14 new *B. infantis* strains (Table S1). Together with the collection strains also analyzed, a consensus phylogenetic tree of the concatenated MLST data was created (Figure S1). Additional information about the MLST analysis is shown in Tables S2–S5.

**Figure 1:**
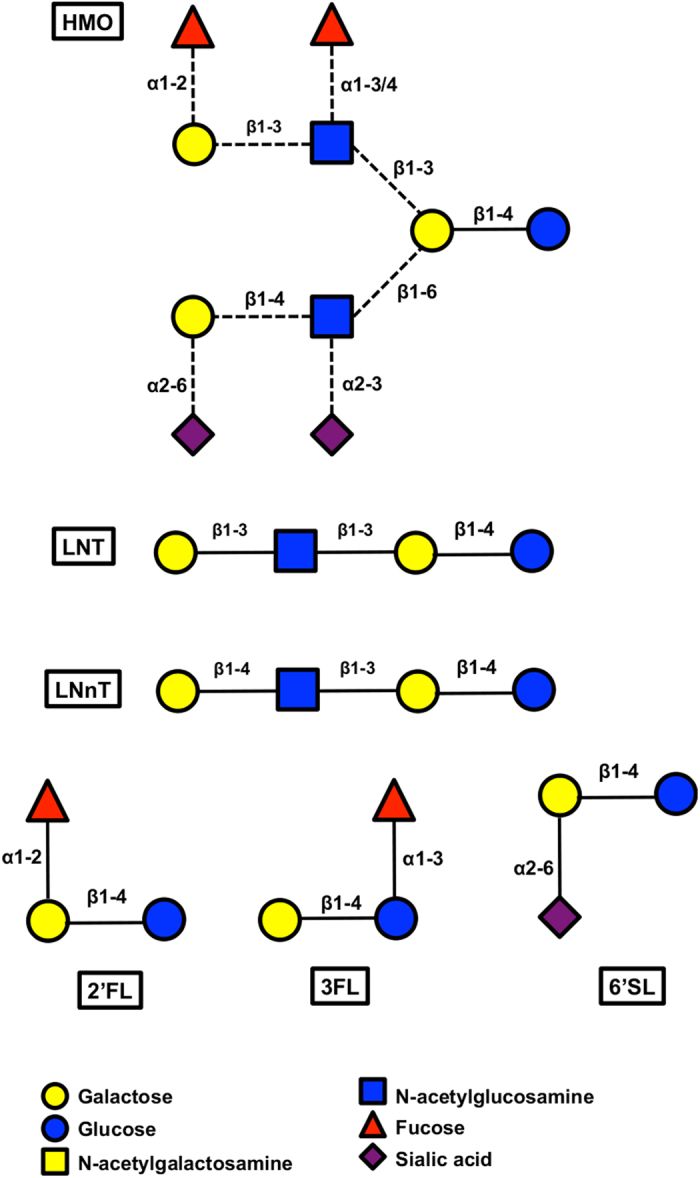
Representative structure of HMO and major oligosaccharides found in breast milk. Dashed lines represent glycosidic linkages not found in all HMO.

Previous studies have characterized the HMO utilization phenotype using pooled HMO purified from breast milk. Here we evaluated the ability of *B. infantis* and *B. bifidum* strains to use individual milk oligosaccharides as a carbon source. We examined strains typed in this study, in addition to other *B. infantis* and *B. bifidum* collection strains^28^ (Table S1). In addition to HMO purified from pooled breast milk samples, we used synthetically generated HMO species such as lacto-*N-*tetraose (LNT), lacto-*N*-neotetraose (LNnT), 2-fucosyllactose (2FL), 3-fucosyllactose (3FL), and 6′sialyllactose (6SL). These individual HMO were selected to represent major structural classes such as neutral non-fucosylated HMO (LNT and LNnT), fucosylated HMO (2FL and 3FL), and sialylated HMO (6SL). The structures of these HMO species are depicted in Fig. 1. In addition, all strains were tested for growth on porcine mucin, which is a glycosylated protein accesible to certain gut microbes^29^.

In general, all *B. infantis* strains grew to high OD_600_ values on pooled HMO and every individual HMO tested (Table 1). One exception is the utilization pattern of 6SL, where in a few cases moderate or poor growth was observed. In contrast, mucin was not a utilizable substrate for any *B. infantis* strain, results in agreement with previous observations^30^ for the type strain ATCC 15697 (Table 1).

Interestingly, *B. bifidum* strains exhibited more phenotypic variability while subsisting on HMO (Table 1). Although most strains were capable of utilizing pooled HMO, four of the thirteen strains (JCM 1255, JCM 1209, JCM 7004 and S28a) showed little capacity to grow on pooled HMO, LNT, LNnT or even mucin (Table 1). JCM 1255 is the type strain and it is known to not represent the growth of *B. bifidum* on HMO^31^. Utilization of 2FL, 3FL and 6SL was also strain-dependent, with several strains unable to use 6SL as the sole carbon source (Table 1).

**Figure 2:**
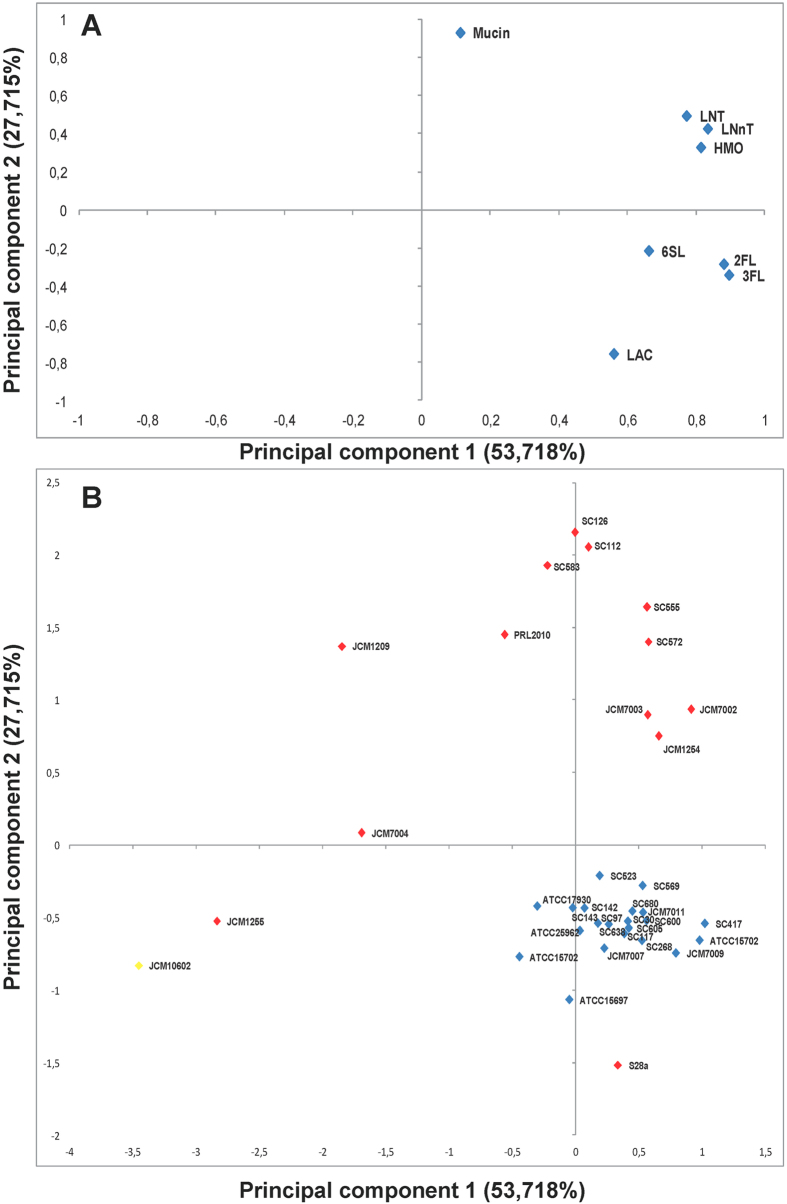
Principal component analysis (PCA) defined by the two principal components (PC1 and PC2) of the maximum OD_600_ values of *B. infantis* (blue) and *B. bifidum* (red) growth on HMO, LNT, LNnT, 2FL, 3FL, 6SL, mucin and lactose. Negative control, *B. animalis* (Yellow). (**A**) represents variables (oligosaccharides substrates) and (**B**) bifidobacteria strains.

We performed a principal component analysis (PCA) with the maximum OD_600_ values of each strain growing on every substrate used in the study (Fig. 2). The descriptors HMO, LNT, LNnT, LAC, 2FL, 3FL and 6SL were mainly explained by the first axis of PCA analysis (Principal component 1) and are clearly associated with most of the bifidobacteria strains studied. The clustering of these variables indicates that *B. infantis* and *B. bifidum* strains grew on HMO similarly to LNT and LNnT. In addition, maximum OD_600_ values on 2FL, 3FL and 6SL were more similar to each other (Fig. 2A). The second axis of PCA (Principal component 2) was defined by mucin growth and was positively correlated with most of *B. bifidum* strains. In addition, *B. infantis* strains clustered more tightly together compared to *B. bifidum* strains, which spread in the plot and displayed more variable growth on HMO included in the analysis (Fig. 2B). Collectively, these results show that the HMO growth phenotype is mostly conserved among *B. infantis* isolates, and that *B. bifidum* displays a larger diversity of phenotypes in HMO consumption.

### Genome sequencing of *B. bifidum* SC555

In order to analyze the global transcriptome of representative strains of *B. infantis* and *B. bifidum* in response to major milk oligosaccharides, we first obtained the genome sequences of representative species. *B. infantis* ATCC 15697 was chosen due to the availability of a completely sequenced genome^22^ and the conservation of genetic and phenotype observed among *B. infantis* strains here and previous studies^28^. We sequenced the genome of *B. bifidum* SC555, a strain isolated from breast-fed infant feces, as it exhibited the best growth on HMO fractions tested in this study. Genome sequencing of SC555 generated over 40 million reads generated on a Illumina HiSeq sequencer. Reads were assembled using the genome of *B. bifidum* PRL2010 as reference^20^, annotated and deposited in the Integrated Microbial Genome Expert Review annotation platform (https://img.jgi.doe.gov, GOLD Project ID: Gi17163).

The draft genome of *B. bifidum* SC555 assembled to 22 contigs with an average length of 101,703 bp (Table S6). The SC555 genome totals 2.24 Mbp, with a G + C content of 62.65%. These values are similar to those observed for other available *B. bifidum* genomes (Table S6). Analysis of orthologous genes shared in strain SC555 and *B. bifidum* PRL2010 indicates a high degree of similarity (Figure S2). In addition, unique genes on each genome are mostly hypothetical without a predicted function, associated with prophages, or restriction-modification systems (data not shown). Finally, *B. bifidum* SC555, PRL2010 and JCM1255 have an identical number of transporters and glycosyl hydrolases previously characterized in the context of HMO utilization^20^,^26^ (Table S7).

### The transcriptomes of *B. infantis* and *B. bifidum* in response to HMO

To profile the global transcriptional response of *B. bifidum* and *B. infantis* to major milk oligosaccharides, two sets of experiments were carried out. First, strains *B. infantis* ATCC 15697 and *B. bifidum* SC555 were grown on HMO purified from pooled breast milk^18^. Samples were extracted from duplicate cultures at early, mid and late exponential phase of growth (referred to as HMOearly, HMOmid and HMOlate). Second, these two strains were cultured on single purified milk oligosaccharides as the sole carbon source: LNT, LNnT, 2FL, 3FL, and 6SL. Sampling was performed at the mid-exponential phase. The two bifidobacterial strains were also grown on lactose as a reference sugar, and *B. bifidum* SC555 was also grown on porcine mucin for comparative purposes.

RNA extracted from the above-mentioned samples were depleted of ribosomal RNA and used to quantify absolute transcript concentrations using RNA sequencing (RNA-seq). It has been recently estimated that 5–10 million reads is an adequate number to interrogate the bacterial transcriptome with high resolution^32^,^33^. RNA-seq experiments generated over 365 million reads in total, with each sample averaging 10.4 million reads for *B. infantis* samples, and 8.8 million reads for *B. bifidum*. Over 70% of these reads aligned to coding genes; 15% aligned to intergenic regions (Table S8). Ribosomal RNA depletion led to total average representation of 16S and 23S rRNA transcripts of less than 1%. General features and statistics from the RNA-Seq experiments are summarized in Table S8.

**Figure 3:**
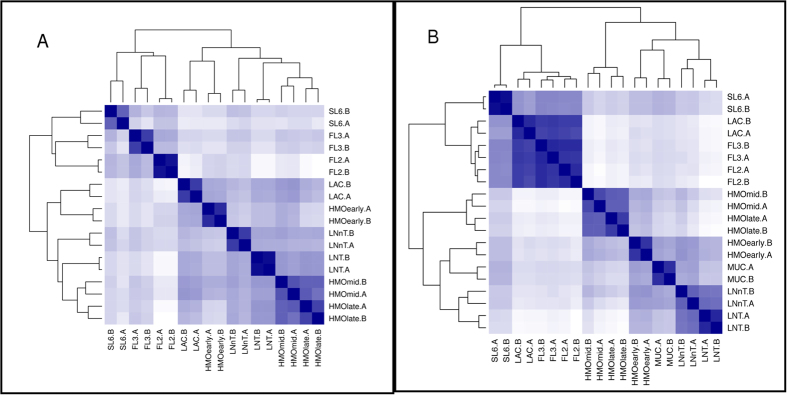
Whole transcriptome distances in substrate responses of *B. infantis* (A) and *B. bifidum* (B). The heatmap shows the distances between the *B. infantis* whole transcriptomes in response to the substrate listed: SL6 = 6′siayllactose, FL3 = 3-fucosyllactose, FL2 = 2′fucosyllactose, HMOearly = HMO at early time point, HMOmid = HMO at mid time point, HMOlate = HMO at late time point, LNT = Lacto-*N-*tetraose, LNnT = Lacto-*N-*neotetraose, “(.A)” = first biological replicate, “(.B)” = second biological replicate. The distances calculated are Euclidian distances of the variance-stabilized count data. The darkness of the blue color in the heatmap indicates the degree of similarity, from white (dissimilar) to dark blue (most similar). Likewise, the dendograms also show the distances with larger branch lengths indicating greater distances.

We first compared the whole transcriptomes of both species on every glycan substrate and growth phase (Fig. 3A,B) using hierarchical clustering. In all RNA-seq experiments conducted on both bifidobacterial species, biological replicates were more similar to each other than to other samples as expected. For *B. infantis*, whole transcriptomes in response to 2FL, 3FL, and 6SL formed one primary cluster that was distinct from lactose (Fig. 3A). In contrast, we found that global transcriptomes of growth on LNT and LNnT were much more similar to HMO (Fig. 3A). In addition, temporal responses to growth on HMO were also similar among each other, indicating that the initial response mounted to HMO is rather stable during exponential growth on HMO.

Unlike *B. infantis*, the global transcriptional responses of *B. bifidum* to 2FL, 3FL, and 6SL were very similar to lactose (Fig. 3B), forming a separate branch in the analysis. Transcriptomes of *B. bifidum* SC555 during growth on HMO at different time points were also closely similar to profiles on LNT, LNnT and also mucin. In summary, for both species global transcriptomes of growth of LNT and LNnT resembled more that of pooled HMO at any point during exponential growth, with smaller HMO eliciting similar transcriptomes to lactose.

### Genes highly expressed in both *B. infantis* and *B. bifidum*

Certain genes were highly expressed regardless of the growth conditions in both *B. bifidum* and *B. infantis* (Tables 2 and 3). These are housekeeping genes and provide insights into bifidobacterial physiology. These genes include ribosomal proteins and elongation factors used in protein synthesis. Interestingly, the most transcribed locus across all samples was RNase P, which is a catalytic RNA that participates in post-transcriptional modification of tRNA molecules^34^. The gene is encoded by BBIF_01522 in *B. bifidum* and what was previously annotated to be an intergenic region in *B. infantis*^22^ (coordinates 1,013,344–1014356). Several genes encoding subunits of the F_1_F_0_ ATP synthase were also highly expressed (Tables 2 and 3). Other genes, such as a bacterial histone-like protein (Blon_0679 and BBIF_00999), chaperonines, and key enzymes of the bifid shunt such as fructose-6-phosphate phosphoketolase (Blon_1722 and BBIF_00286) and glyceraldehyde-3-phosphate dehydrogenase (Blon_0900 and BBIF_01046) were highly expressed as well.

### Comparative transcriptomics of *B. infantis* in response to different milk oligosaccharides

Comparative genomics and functional studies have previously provided a framework of HMO utilization in *B. infantis* and *B. bifidum*^8^,^20^,^22^,^26^,^35^, which includes ABC transporters and uniporters, glycosyl hydrolases and feeder pathways. Lists including these genes and their annotations are presented in Tables S9 and S10. In addition to confirming expression of genes of interest during growth on HMO, RNA-seq was used to characterize change in expression in response to specific HMO structures and how these responses evolve during exponential growth.

**Figure 4:**
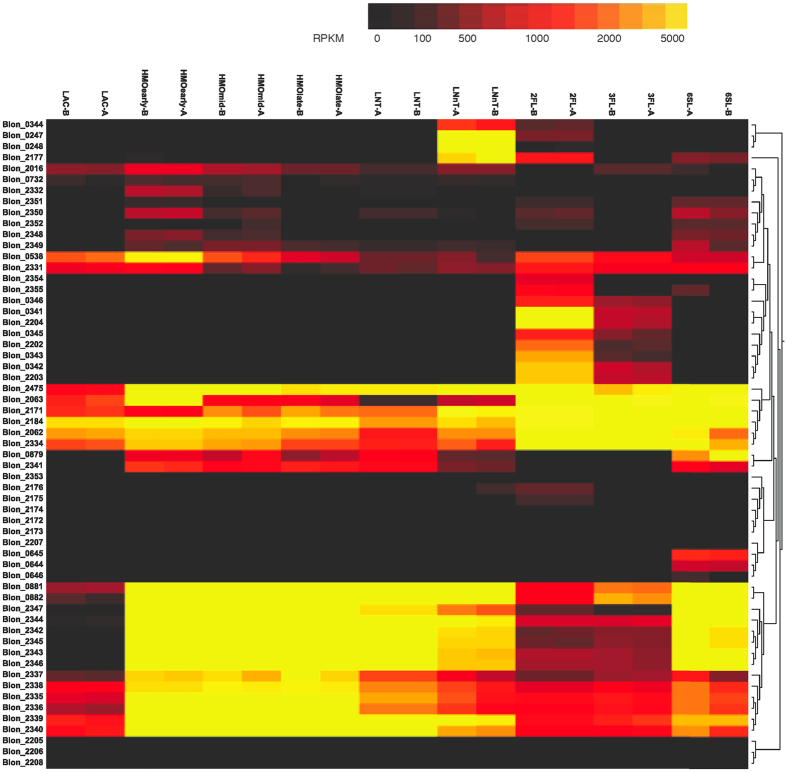
Hierarchical clustering of features (HCF), of the whole transcriptome of *B. infantis* during growth on HMO and major HMO. The heat map represents the expression of genes in Table S9.

A first subset of genes displayed consistent expression values across all conditions tested (Fig. 4). These genes encode important functions such as an ATPase associated to ABC transporters (Blon_2475), galactose metabolism genes and a β-galactosidase with lactase activity^36^, Blon_2334 (Fig. 4 and Table S9).

Among genes associated with HMO consumption in *B. infantis* (Table S9), RNA-seq confirmed that several of them are significantly upregulated during growth on HMO (Fig. 4). This induction was steady across exponential growth time points (Fig. 4). These genes are located mostly in the HMO cluster I^22^ (Blon_2335 to Blon_2346), and Blon_0881 and Blon_0882, encoding enzymes responsible for GlcNAc derivation to the bifid shunt. These results suggest that the HMO cluster I acts as an HMO-inducible unit, and that it is co-regulated in a similar fashion with this GlcNAc utilization pathway.

It is interesting to note that these HMO-utilization genes are also induced several fold when *B. infantis* grows on LNT, LNnT and 6SL (Fig. 4). While this is expected for LNT, which is the most abundant HMO^5^, and for LNnT which is an isomer of LNT, 6SL eliciting a similar response to HMO was not predicted. LNT and LNnT are GlcNAc containing neutral HMO, while 6SL contains a negatively charged sialic acid residue.

In contrast, the expression of these genes is different when *B. infantis* consumes 2FL or 3FL (Fig. 4). When growing on these sugars as the sole carbon source, the expression of the HMO cluster displayed only basal RPKM values, and other gene clusters appear to be used instead. Genes Blon_0341 to Blon_0344, Blon_2202 to Blon_2204 and Blon_2305 to Blon_2309, were induced by 2FL, 3FL but no other substrates. These genes encode functions related to FL import through ABC transporters^23^, and putative enzymes participating in fucose metabolism^37^ (Table S9). Thus, the transcriptional results suggest that fucosyllactose is imported and catabolized using proteins encoded by these alternative genes.

### Differential transcription of key functions of the HMO utilization in *B. bifidum*

A model has been proposed regarding how *B. bifidum* obtains energetic value from HMO and mucin glycoconjugates^20^,^32^. Important determinants are extracellular glycosyl hydrolases, that generate mono and disaccharides that are captured by membrane porters and one ABC transporter, specific for HMO-derived lacto-*N-*biose (LNB) and mucin-derived galacto-*N-*biose (GNB). This gene set is shown in Table S10.

**Figure 5:**
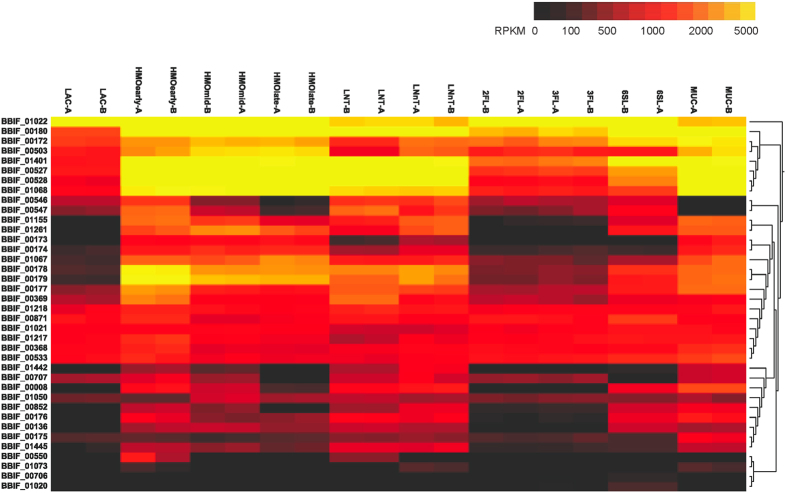
Hierarchical clustering of features (HCF), of the whole transcriptome of *B. bifidum* during growth on HMO and major HMO. The heat map represents the expression of genes in Table S10.

Some of these genes displayed high expression at all time points during growth on HMO, regardless of the substrates used (Fig. 5). These include important determinants in *B. bifidum* HMO and mucin consumption, such as lacto-*N-*biosidase (BBIF_00533), lacto-*N-*biose phosphorylase (BBIF_01217), and an endo-α-*N-*acetylgalactosaminidase (BBIF_00503).

Several of these genes were largely induced during growth on HMO compared to lactose. In general, this response was stronger during early growth, with lower values to the end of exponential growth, including the LNB/GNB ABC transporter cassette and the LNB/GNB utilization pathway (Fig. 5, Table S10). Interestingly, these genes were similarly induced during growth on LNT, LNnT and even mucin, likely reflecting the importance of LNB and GNB to *B. bifidum*^38^.

In contrast, the LNB/GNB cluster and pathway were not induced by 2FL or 3FL, probably indicating that *B. bifidum* SC555 is mostly unresponsive to these substrates (Fig. 5). This is in agreement with the observation that two genes encoding α-fucosidases (BBIF_00008 and BBIF_01261), are actually induced by HMO, LNT, LNnT and mucin, but not by 2FL or 3FL (which contain fucose). Collectively, these results suggest that while *B. bifidum* can use larger fucosylated oligosaccharides, it does not show a preference for 2FL or 3FL and it only benefits from the lactose on these oligosaccharides.

### Indirect comparison of *B. infantis* and *B. bifidum* by functional enrichment analysis

In a different set of analyses, whole transcriptional responses for both bifidobacterial species were compared indirectly by annotating all genes by function using Clusters of Orthologous Groups (COG) of proteins functional categories, gene ontology (GO) terms, Kyoto Encyclopedia of Genes and Genomes (KEGG) pathways, and Stanford Research Institute (SRI) pathways^54^. We next determined which of these predicted functions were differentially enriched within and between the two species (Tables S11–S15).

The two species displayed different functional enrichments when examining genes up-regulated in the presence of HMO at the initial sampling point. During the HMOearly time point relative to lactose, the *B. infantis* transcriptome is enriched for the GO terms “carbohydrate transport” and “transporter activity” as well as COGs of both components of the ABC-type sugar transport system (Table S11). This enrichment was also observed during growth on individual HMO, including 2FL and 3FL (Table S11). In contrast, the *B. bifidum* transcriptome was not enriched for “carbohydrate transport” until HMOlate samples (Table S13). Instead, genes upregulated by *B. bifidum* at the HMOearly time point were enriched for translation (e.g. ribosome, rRNA binding, translation) as well as “cell adhesion” and “carbohydrate metabolic process” (Table S13). GO terms and COGs related to carbohydrate transport were also enriched in the *B. infantis* response to individual HMO substrates, but not in the *B. bifidum* response to these substrates (Tables S11 and S13). Interestingly, *B. infantis* appears to down-regulate nucleotide biosynthesis, at the mid and late phase of growth on HMO as well as on LNT relative to lactose. These observations support our hypothesis that *B. infantis* has innovated several parallel systems to transport intact milk oligosaccharides. This is in contrast with *B. bifidum* which deploys a far more limited oligosaccharide transport system with a substrate acquisition strategy focused on extracellular mono- and disaccharides generated from HMO.

### Direct comparison of *B. infantis* and *B. bifidum* by orthologs

To directly compare the transcriptional response between the two bifidobacterial species, we identified orthologs for which there is a single copy present in each genome (see Methods). Subsequently, we placed all gene expression values on this common coordinate system. Between the *B. infantis* and *B. bifidum* genomes, 1266 single copy orthologs were identified. Of these, between 772 and 1113 were differentially expressed in the two species in response to the same substrate (Table S15). In response to each substrate, roughly half of the differentially expressed genes are up-regulated in *B. infantis*, relative to *B. bifidum*, and the other half are up-regulated in *B. bifidum*, relative to *B. infantis* (Table S15). Importantly, each species had a distinct response to each substrate, even lactose, when comparing the orthologous genes.

Even in situations where the two species have the same genetic capacity (i.e. both have a single copy ortholog), the expression of that single ortholog in response to the same substrate varied by nearly 150-fold. For example, components of the phosphotransferase system exist in both species but are up-regulated in *B. bifidum* 139- to 148-fold compared to *B. infantis* in response to HMO at the early time point (Table S16). Other orthologs are likewise highly expressed in one species and essentially unexpressed in the other (Tables S16 and S17). The NLPA lipoprotein (Blon_1721) in *B. infantis* is 130-fold higher than its ortholog in bifidum (BBIF_01591). It is most highly expressed in response to lactose; thus, its function is expected to be independent of HMO substrates.

The *B. infantis* short-chain dehydrogenase/reductase (SDRs) Blon_2339 and Blon_2308, together, are expressed 111.9-fold higher than their orthologous gene in *B. bifidum*, BBIF_01220 (Table S17). These genes are probably involved in fucose metabolism^37^, and are up-regulated in response to HMO relative to lactose (p < 2.3e-31). Blon_2308 is uniquely significantly higher, by over 900-fold, in response to 2FL relative to lactose (p = 0). These data support the idea of fucose being actively metabolized by *B. infantis*, while *B. bifidum* seems to prefer other monosaccharides such as Gal, GlcNAc and Glc, cross-feeding fucose and sialic acid to other species.

**Figure 6:**
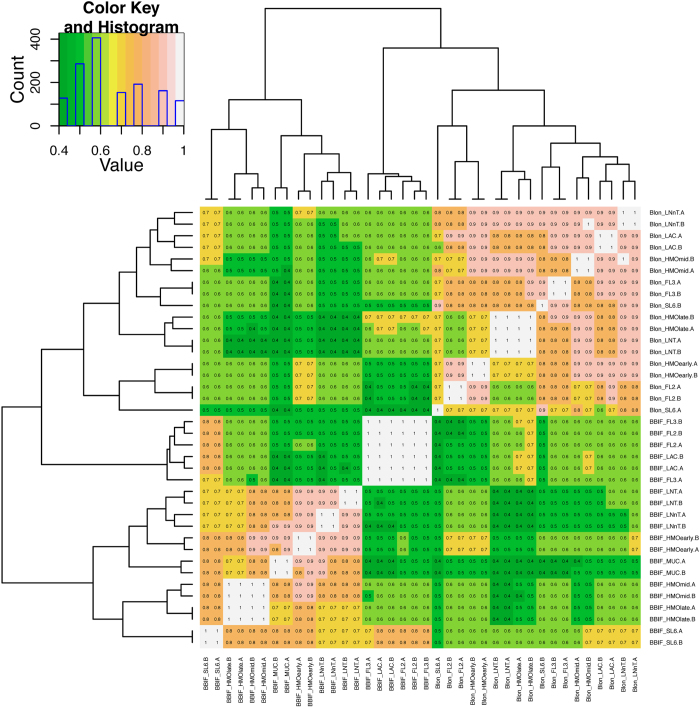
Map of correlations between orthologous transcriptomes in *B. infantis* and *B. bifidum*. Each box in the heatmap represents the correlation between the transcriptomes indicated on the x-axis and y-axis: BLON = *B. infantis*, BBIF = *B. bifidum*, SL6 = 6′siayllactose, FL3 = 3′fucosyllactose, FL2 = 2′fucosyllactose, HMOearly = HMO at early time point, HMOmid = HMO at mid time point, HMOlate = HMO at late time point, LNT = Lacto-*N-*tetraose, LNnT = Lacto-*N-*neotetraose “(.A)” = first biological replicate, “(.B)” = second biological replicate. The color key indicates the correlation also indicated by the text in each box: The color key also includes a light blue line that indicates the number of observations at each level of expression.

A correlation map of the whole transcriptomes, based on single copy orthologs, for both species on all tested substrates is shown in Fig. 6. Perhaps most striking, the *B. bifidum* response to lactose, 2FL, and 3FL is more similar to *B. infantis* HMO-late than it is to its own response to HMO, LNT, LNnT, or mucin (Fig. 6). In other words, the *B. bifidum* response to lactose, 2FL and 3FL correlates poorly with its own response to LNT, LNnT, mucin, or HMO at any point. Relative to lactose, there are only 230 and 138 genes differentially expressed by *B. bifidum* in response to 2FL or 3FL, respectively. In contrast, there are over a 1000 genes differentially expressed by *B. bifidum* in response to the other substrates (Tables S18 and S19). This confirms that *B. bifidum* has a fundamentally different strategy for uptake and utilization of lactose and FL compared to the more complex oligosaccharides.

## Discussion

In this study we compared the physiological and transcriptional responses to HMO of two representatives of important species of infant-gut associated bifidobacteria: *B. infantis* and *B. bifidum*. Importantly, we determined the transcriptional responses to pooled and single isolated HMO molecules, linking in detail biological function with substrate structural diversity.

While almost all *B. infantis* strains were efficient in utilizing a diversity of HMO as the sole carbon source, *B. bifidum* strains were more variable (Table 1). This is interesting since most of the strains studied were from infant origin, and it could be expected that they are all competitive in HMO consumption. While this was the case for *B. infantis*, several *B. bifidum* strains did not show a major preference for HMO, indicating that they may target other carbon sources. Growth analysis in Table 1 showed that medium and poor HMO-utilizing strains still display the ability to grow on LNT or mucin. It is very interesting to note that key HMO genes (lacto-N-biosidase, galactosidases, fucosidases and sialidases; Table S7), are present in the same numbers in high growers (SC555) and poor growers (JCM 1255), indicating that the physiological differences among these strains are due to point mutations or differential regulation of these genes. In addition to the implications to host-microbial interactions, this wide array of responses to HMO has the potential to impact applications such as probiotic design, where strain-level differences in substrate utilization require additional consideration. Another recent study studied a similar number of *B. breve* strains, showing that certain of these isolates were much more competitive in different HMO consumption than previously reported for the type strain^21^.

In this study we used RNA-sequencing for determining the global transcriptome of *B. infantis* and *B. bifidum* during the *in vitro* consumption of specific, structurally different HMO, and during exponential growth. While our group and others have determined certain genes participating in HMO consumption in both species, in this study we were able to screen how individual HMO elicit differential transcriptomes in both bifidobacteria.

An important observation is that *B. infantis* and *B. bifidum* have a markedly different physiological response to FL. Genetic factors (secretor genes) such as the α1,2 fucosyltransferase (FUT2) and α1,3/4 fucosyltransferase (FUT3) genes can significantly alter HMO composition^39^,^40^, especially increasing the abundance of 2FL and α1-2 fucosylated HMO. The impact of milks higher or lower in 2FL on bifidobacterial species is still not clear, although certain studies suggest a correlation between secretor status and the overabundance of certain bifidobacterial species^41^.

In *B. infantis*, we have observed that 2FL or 3FL induces the expression of discrete, alternative operons, distinct from the HMO cluster I. They contain functions related to fucosylated HMO import and fucose utilization, and interestingly are not induced by pooled HMO or single neutral HMO. In contrast, global transcriptomes of LNT and LNnT in both species were highly similar to HMO, and responses to HMO were mostly steady during *in vitro* growth. Noteworthy, several HMO species such as LNT or LNnT enhanced the expression of several HMO consumption genes in a similar manner as pooled HMO. This was evident in the acidic 6SL as well. It is possible that the transcriptomes in response to neutral HMO and 6SL are similar considering that sialic acid and GlcNAc metabolic feeder pathways are convergent in *B. infantis*^31^. Neu5Ac is metabolized to GlcNAc-6-P and later to fructose-6-P in *Escherichia coli* and *Bifidobacterium breve*^42^,^43^, and the respective genes were upregulated during growth on these substrates (Fig. 4, Table S9).

This transcriptional analysis was also useful in providing additional detail on both mechanistic models of HMO consumption deployed by infant-associated bifidobacteria. The similarity of transcriptomes in response to 2FL, 3FL, 6SL and lactose suggests that *B. bifidum* SC555 appears to prefer the utilization of lactose when growing on short HMO, probably releasing fucose and sialic acid to the environment. This in turn might allow cross-feeding to other gut species. This has been recently shown using mucin oligosaccharides and 3SL^43^,^44^, where *B. breve* can feed on sialic acid released from *B. bifidum* activity. This is supported by the absence or lack of activation of fucose and sialic acid metabolic pathways in the genome or transcriptome of *B. bifidum* SC555 (Table S10). In contrast, *B. infantis* seems to activate feeder pathways for fucose and sialic acid (Fig. 4 and Table S9) during most of the conditions tested, reflecting its ability to further process these monosaccharides.

Finally, global transcriptome analysis and ortholog comparison presented another set of fundamental differences between each species’ physiology. While we expected to observe an induction of carbohydrate transport and metabolism genes in *B. infantis*, this upregulation was just modest in *B. bifidum* SC555 especially during growth on FL (Tables S16 and S17). We also noted that expression of several orthologous genes was also drastically different, which indicates that both species operate and respond differently to HMO. For example, while the HMO utilization strategy in *B. bifidum* relies in part on a PTS system, the corresponding homolog in *B. infantis* does not appear to participate in this process. Putative fucose metabolism genes were in contrast induced several fold only in *B. infantis*.

The differences presented therefore reflect two different lifestyles found in the infant intestinal microbiome that have apparently converged in specialization in utilization of HMO. It could be possible that both *B. bifidum* and *B. infantis* display biogeographical differences as well, considering that most *B. bifidum* strains are competitive in mucin degradation, associating this species with the mucus layer. In contrast, *B. infantis* could reside mostly in the lumen. Certain species in the gut microbiome, such as *Clostridium* and *Bacteroides*, can be preferentially located in the mucus layer or the intestinal lumen^45^.

## Conclusions

Both *B. infantis* and *B. bifidum* are able to utilize HMO as a sole carbon source, but with divergent strategies. In the present study, we showed that *B. infantis* strains are highly homogeneous in their ability to use specific HMO substrates, while *B. bifidum* isolates were more diverse, with some strains unable to grow on fucosylated or sialylated HMO. Choosing representative strains that grow well on HMO, we confirm that *B. infantis* expresses an assortment of transporters to internalize HMO substrates while *B. bifidum* expresses enzymes to hydrolyze HMO extracellularly. The transcriptome of each isolate varies dramatically even in response to structurally similar substrates. Likewise, the two strains have different transcriptional responses to the same substrate, even when lactose is the sole carbon source.

Our findings highlight the genetic and functional diversity between strains and between different isolates of the same strain as well as the specific mechanisms used by these isolates to utilize HMO. Such detailed mechanistic knowledge of the relationships between substrates and specific bifidobacteria will inform effective prebiotic and probiotic strategies. Synbiotic supplements could be designed to pair specific prebiotics with probiotics that will consume them via known strategies to achieve predefined functional outcomes.

## Methods

### Bacteria and media

*Bifidobacterium* strains used in this study (Table S1) were obtained from fecal samples from exclusively breast-fed term infants^21^, the Japanese Collection of Microorganisms (Riken Biosource Center Japan), the American Type Culture Collection (Manassas, VA), and the University of California Davis Viticulture and Enology Culture Collection (Davis, CA). For routine experiments, bifidobacteria were grown on de Mann-Rogose-Sharp (MRS) broth supplemented with 0.05% w/v L-cysteine (Sigma-Aldrich, St. Louis, MO), and incubated for 18 h at 37 °C in an anaerobic chamber (Coy Laboratory Products, Grass Lake, MI), in an atmosphere containing 5% carbon dioxide, 5% hydrogen, and 90% nitrogen. Prior to each assay all bacteria were subcultured twice.

### Multilocus sequence typing (MLST) of strains

MLST analysis of *B. infantis* and *B. bifidum* strains targeted intragenic regions of seven housekeeping genes *clpC, purF, gyrB, fusA, Iles, rplB, rpoB* that were selected based on a previous study^27^. For PCR amplification, one μl of extracted DNA was added to 50 μl reaction mixture containing 50 pmol of primers, 200 μM of each dNTP, 0.1 vol of 10X PCR buffer, 2.5 mM MgCl2, and 1 U AmpliTaq gold polymerase (Applied Biosystems). Cycling conditions were optimized for every primer set (Table S2) and consisted of an initial denaturation at 95 °C for 4 min, followed by 35 cycles of 95 °C for 30s, annealing at 60–67 °C for 30s, elongation at 72 °C for 60s, final extension at 72 °C for 7 min, and holding at 4 °C. The resulting amplicons were separated using a 1% agarose gel, followed by GelRed staining (Phenix Research Products, Candler, NC), and purification using a QIAquick PCR Purification Kit (Qiagen, Valencia, CA). Sequencing was performed on an ABI 3730 Capillary Electrophoresis Genetic Analyzer using BigDye Terminator chemistries at the University of California Davis DNA Sequencing Facility. Sequencing data for all loci was edited using BioEdit 7.0 and aligned using CLUSTAL W^46^. Phylogenetic analysis and concatenations of the sequenced loci were performed using the Molecular Evolutionary Genetic Analysis (MEGA) software version 5 (http://megasoftware.net). Descriptive evolutionary analysis including mol% G + C content, number of polymorphic sites, nucleotide diversity π/site, average number of nucleotide differences k were calculated using DnaSP version 5.10 (Table S3). Allelic sequences were assigned as described previously^47^ (Tables S4 and S5). A minimum evolution tree of the concatenated loci was calculated using MEGA 5.0 (Fig. S1).

### Bifidobacterial growth *in vitro* on HMO

The 35 bifidobacterial strains in Table S1 were tested for growth in the presence of seven different substrates: HMO^18^, LNT, lacto-*N*-neotetraose (LNnT), 2′-fucosyllactose (2FL), 3-fucosyllactose (3FL) (Glycom, Denmark), 6′-sialyllactose (6SL) (GenChem Inc. Korean), and hog mucin type II (Sigma). *B. animalis subsp. lactis* JCM 10602 was also included as negative control for growth experiments. Two μl of each resulting overnight culture were used to inoculate 200 μl of modified MRS medium (mMRS), devoid of glucose and supplemented with 2% (w/v) of each substrate, except for mucin at 1%, as the sole carbohydrate source, and another 2 μl inoculated into mMRS without added sugar. Mucin was autoclaved at 121 °C for 10 minutes before addition to broth. The media was supplemented with 0.05% (w/v) L-cysteine, and in all the cases the cultures in the wells of the microtiter plates were covered with 30 μl of sterile mineral oil to avoid evaporation. The incubations were carried out at 37 °C in an anaerobic chamber (Coy Laboratory Products, Grass Lake, MI). Cell growth was monitored in real time by assessing optical density (OD) at 600 nm using a BioTek PowerWave 340 plate reader (BioTek, Winoosky, VT) every 30 min preceded by 15 seconds shaking at variable speed. Two biological replicates and three technical replicates were performed for every studied strain. The OD obtained for each strain grown on the different substrates, was compared with the OD obtained in the absence of a sugar source. This difference in OD (ΔOD) was used as a parameter to evaluate each strain’s ability for growing on the different substrates.

### Statistical analysis of growth

Statistical analysis of the data was carried out using SPSS for Windows, 15.0 (SPSS Inc Chicago, IL, USA). The relationships among the maximum OD_600_ values of the growth on the different substrates by the bifidobacteria strains were evaluated by Pearson correlation coefficients and principal components analysis (PCA).

### Genome sequencing

*B. bifidum* SC555 is a strain that was previously isolated from a 3–6 month old infant. For genome sequencing, the strain was grown on MRS broth under the anaerobic conditions described above. After 18 h, 5 ml of this culture was centrifuged at 12000 × *g* for 2 min, and used to extract DNA using the MasterPure Gram-positive DNA Purification Kit (Epicentre), following manufacturer instructions. DNA was quantified using the dsDNA HS Assay in a Qubit 2.0 fluorometer (Life Technologies, Carlsbad CA). DNA was also checked for integrity in 1% agarose gels. Next, 2 μg of the genomic DNA in 100 μl of elution buffer EB were sonicated in a Bioruptor Standard (Diagenode, Denville NJ), for 10 min with cycles of 30s on and 30s off. Samples were stored at −20 °C, and fragmentation sizes were evaluated in a 2100 Bioanalyzer using the Agilent DNA 1000 kit (Agilent Technologies, Santa Clara CA). Fragmented cDNA (15 μl at 20 ng/μl) was used to prepare a library for Illumina sequencing using the Apollo 324 Robot and the PrepX ILM DNA Library Kit (IntegenX, Pleasanton CA), at the Genome Center at UC Davis and following manufacturer instructions. Sample was ligated with BiooScientific Adapter 15 (Bioo Scientific, Austin TX). The resulting library was checked for size using a High Sensitivity DNA Kit (Agilent) in the Bioanalyzer 2100, with an expected average size of 350 bp. Finally, sample was pooled with other seven bacterial genomes at the Genome Center at UC Davis DNA Technologies Core Facility, for sequencing on a Illumina HiSeq2500. All sequencing steps were carried out by Core personnel. Sequencing was run for 200 cycles, with read length of 100 bp (paired ends).

Sequencing files were concatenated using Terminal, and processed for genome assembly using CLC-Bio Genomics Workbench (Cambridge, MA). Reads (100 bp) were trimmed setting at 2 the maximum of ambiguous base pairs, and deleted if their quality scores were below 0.5. Edited reads were next aligned to the genome sequence of *Bifidobacterium bifidum* PRL2010. Genbank files were obtained from the Integrated Microbial Genome database^48^. Resulting contigs from the genome of *B. bifidum* SC555 were submitted to the IMG Expert Review (IMG/ER) for annotation (GOLD card: Gi17163. ER submission ID: 8631). Locus tags generated contained the BBIF prefix, and topology was linear. Gene calling method was the Isolate Genome Gene Calling. Draft genome sequences were analyzed using the IMG Expert Review functions. For creating a Venn diagram, Genome Gene Best Homologs between *Bifidobacterium bifidum* SC555 and PRL2010 was used. Analysis was set to look for homologs using BLASTP with more than 60% identity at the aminoacid level. The final draft genome of *Bifidobacterium bifidum* SC555 is available at https://img.jgi.doe.gov, GOLD Project ID: Gi17163.

## RNA-seq

### Sample preparation, growth curves

*B. longum subsp. infantis* ATCC 15697 *and B. bifidum* SC555 were grown as described above in mMRS supplemented with either 2% lactose, 2% HMO, 2% LNT, 2% LNnT, 2% 2FL, 2% 3FL, 2% 6SL or 1% porcine mucin in a microplate reader, and cultures were taken at mid-exponential phase OD 0.5–0.6. In the case of HMO, the samples were collected at three different points in the growth curve, approximately OD_600nm_ = 0.25 (early exponential), 0.5–0.7 (mid exponential) and 0,9-1.1 (late exponential). Samples were immediately pelleted at 12000 × *g* for 1 min and stored in RNAlater Ambion. RNA extraction, and cDNA conversion were performed as previously described^23^.

### RNA extraction

One ml of each sample stored in RNAlater, with OD above 0.6, was used in the procedure. Samples were centrifuged at 10,000 × *g* for 2 min on a bench centrifuge to collect the cell pellet. Pellet was washed twice with PBS buffer to remove RNAlater, and centrifuged at 10,000 × *g*. Pellets were later resuspended in 250 μl of lysozyme (50 mg/ml final; Sigma) and 125 μl of mutanolysin (1000 units/ml; Sigma), previously prepared and filter-sterilized in TE buffer (10 mM Tris, 1 mM EDTA, pH 7.2). Samples were incubated at 37 °C for 10 min. Pre-lysed cells were centrifuged at 8000 × *g* for 1 min, and the Ambion RNAqeous kit (LifeTechnologies) was used to extract total RNA following manufacturer instructions and eluting the RNA in 50 μl of EB buffer. Total RNA was immediately subjected to DNase treatment with the Turbo DNase free (Ambion) using 3 μl of DNase I for 1 hour. RNA was stored at −80 °C at this point, but previously quantified in Qubit using the Qubit High Sensitivity RNA Assay Kit (Life Technologies). One μl of RNA was the minimum used in next steps. RNA integrity was checked in a Bioanalyzer using an Agilent RNA Nano Chip. RNA (1–2 μl) was first denatured at 65–70 °C for 2 min before the run. Samples were further processed only if their RIN (RNA Integrity Number), was above 7. We later evaluated genomic DNA contamination by qPCR, using the Fast Sybr-Green Master Mix (Applied Biosystems, Foster City CA), and universal bacterial primers Uni334F (5′ACTCCTACGGGAGGCAGCAGT) and Uni514R (5′ATTACCGCGGCTGCTGGC). Template DNAs were the RNA samples before and after DNAse treatment, and proper negative and positive controls were used. Each reaction (20 μl total) included 1X master mix, 0.1 μm primers, and 1 μl RNA. Samples were run in triplicate.

Ribosomal RNA depletion was achieved using the Ribo-Zero magnetic kit, Bacteria (Epicentre, Madison WA). Manufacturer instructions were followed (using 28 or 26 μl of RNA depending on the concentration). mRNA was purified with the Qiagen RNeasy Minelute Cleanup kit, following the instructions presented in the Ribo-Zero protocol for this part. RNA was eluted in 13 μl. One μl of the RNA was checked for removal of 16S and 23S rRNA peaks using the Qubit HS RNA Assay (Agilent). Chromatograms were manually inspected.

mRNA was later converted to cDNA. For first strand cDNA conversion, the Superscript II Reverse Transcriptase was used (Invitrogen), following manufacturer protocol. Reaction also contained Random Primers (3 μg/μl, Invitrogen). We used 11 μl of rRNA depleted RNA, 1 μl of 10 mM dNTPs and 1 μl of random primers. After this, second cDNA strand was synthesized using the NEB NEBNext mRNA Second Strand Synthesis Module (New England Biolabs, Ipswich, MA) following manufacturer instructions. Then, the MinElute Reaction Cleanup Kit (Qiagen, Valencia CA) was used for cleaning up the DNA. Final elution was done in two rounds of 15 μl in EB buffer. At this point cDNA was quantified using the Qubit High Sensitivity DNA kit (Life Technologies), and diluted to a volume of 100 μl with EB buffer, for fragmentation in a Bioruptor (Diagenode). Eight cycles of 15s on and 90s off were used for each sample. Fragmented cDNA was quantified with Qubit High Sensitivity DNA kit. DNA was also analysed in a Bioanalyzer with the Agilent High Sensitivity DNA kit. An average fragment length of 300–500 bp was desired for next steps.

### Library construction

Sequencing libraries were prepared using the BiooScientific NEXTflex Chip Seq Kit (Bioo Scientific, Austin TX). This kit is Illumina compatible. Ten ng of fragmented cDNA were used. In the manufacturer protocol, option 3 was followed entirely, with the goal to select fragments between 300 and 400 bp in a gel-free protocol. Size selection and clean-up steps were achieved using the Agencourt Ampure XP beads (Beckman Coulter, Brea CA), and a Magnetic Stand-96 (LifeTechnologies). All temperature-sensitive reactions were carried out in a preheated thermocycler. Adapters used (NEXTflex™ ChIP-Seq Barcodes, Bioo Scientific) contained 6-bp indexes for multiplexing. At the last step, ligation products were amplified by PCR with the supplied reagents for 17 cycles.

Libraries were first assessed in Bioanalyzer using a DNA high sensitivity Agilent chip. Sizes were expected to be in the 300–500 bp range. Libraries were quantified using the Kapa SYBR FAST Universal qPCR Kit. At maximum, 24 libraries with different barcodes were pooled in one reaction tube containing 20 ng of each library. The library was further assessed in an Agilent High Sensitivity DNA kit and quantified by Qubit HS DNA assay. Pooled libraries were submitted to the Genome Center at UC Davis Genome Center DNA Technologies Core Facility, for sequencing on a Illumina HiSeq2500. All sequencing steps were carried out by Core personnel. Sequencing was run for 50 cycles, with read length of 50 bp (single reads). Runs were demultiplexed by the Core Facility.

Received files were first joined using the Terminal software, and exported to the CLC-Bio Genomics Workbench. This platform was used in all analyses unless mentioned. Reads (100 bp) were trimmed setting at 2 the maximum of ambiguous base pairs, and deleted if their quality scores were below 0.5. Edited reads were aligned to fasta files generated at IMG from the genome sequences (reference) of *B. longum* subsp. *infantis* ATCC 15697, and *B. bifidum* SC555, respectively. Only coding regions were considered in this analysis. Alignment allowed a maximum of 2 mismatches, and a maximum of 10 matches to other reads. Other settings were considered as default. The CLC-BIO tools retrieved information regarding the gene length, total and unique gene reads aligning to a specific locus tag, and automatically calculated the RPKM value (Reads Per Kilobase per Million mapped reads)^49^.

### Statistical analysis

RPKM information for each gene in each genome was used in CLC-Bio Genomics Workbench for statistical comparisons, under the Set Up Experiment option. This included each sample corresponding to *B. infantis* or *B. bifidum* growing on a different substrate or growth phase. Samples were considered as independent or unpaired (multi-group comparison). RPKM values were transformed to log10 for statistical analysis. In box plots, the box is defined by the inter quartile ranges (IQR), the median is drawn as a line in the box, and the whiskers extend 1.5 times the height of the box. Principal component analysis is presented on transformed values for each sample, including the biological replicates. Statistical analyses were carried out on proportion-based Baggerly test^50^. Samples from growth on lactose were considered as reference controls. p-values were applied the Bonferroni correction. Finally, hierarchical clustering of features was calculated on original RPKM values, considering Euclidean distance and Single cluster linkage.

### Differential expression

To determine differentially expressed genes, the R package “DESeq^51^” was applied to the raw count data. Genes with a mean count less than 200 were excluded from analysis. Adjusted p-values <= 0.05 were deemed statistically significant. Where a singular p-value has been stated in the manuscript for a group of tests, the highest (least significant) p-value is reported.

### Functional enrichment analyses

For each substrate group, genes were ranked by their log2foldchange and p-value for gene expression relative to lactose. Those with a non-significant p-value received a rank of 0 (effectively ending up in the middle of the list) while those with a significant p-value received a rank equal to their log2 fold change. Up-regulated genes appear towards the top of the list while down-regulated genes appear toward the bottom of the list. The ranked gene lists were analysed using Gene Set Enrichment Analysis (GSEA)^52^ for enrichment with functional annotations towards the top of the list (e.g. those most differentially expressed). Custom scripts were developed to create GSEA input files for different functional annotations. Functional annotations tested included COG functions, KEGG pathways, GO terms, and SRI pathways. GO annotations were derived for each gene using Blast2GO^53^. SRI pathway annotations were mapped using SRI Pathway Tools^54^.

## Availability of supporting data

The *Bifidobacterium longum subsp. infantis* ATCC 15697 genome used in this manuscript is available in the Integrated Microbial Genomes database at the Joint Genome Institute at https://img.jgi.doe.gov, GOLD Project ID: Gc00886. The *Bifidobacterium bifidum* SC555 genome is also available at https://img.jgi.doe.gov, GOLD Project ID: Gi17163. The data from the *B. infantis* and *B. bifidum* RNA-sequencing studies are available in the NCBI Gene Expression Omnibus database (http://www.ncbi.nlm.nih.gov/geo/) with accession numbers GSE58773 and GSE59053, respectively.

## Additional Information

**How to cite this article**: Garrido, D. *et al.* Comparative transcriptomics reveals key differences in the response to milk oligosaccharides of infant gut-associated bifidobacteria. *Sci. Rep.* **5**, 13517; doi: 10.1038/srep13517 (2015).

## Supplementary Material

Supplementary Information

## Figures and Tables

**Table 1:** Level of growth on different glycans by *B. bifidum* and *B. infantis* strains.

Strains	HMO	LNT	LNnT	2FL	3FL	6SL	Mucin	Lactose
*B. infantis*								
ATCC 15697	+++	+++	+++	+++	+++	+++	−	+++
ATCC 25962	+++	+++	+++	+++	+++	+++	−	+++
ATCC 17930	+++	+++	+++	+++	+++	++	−	+++
ATCC 15702	+++	+++	+++	+++	+++	++	−	+++
JCM 7007	+++	+++	+++	+++	+++	++	−	+++
JCM 7009	+++	+++	+++	+++	+++	+++	−	+++
JCM 7011	+++	+++	+++	+++	+++	+++	−	+++
SC30	+++	+++	+++	+++	+++	++	−	+++
SC97	+++	+++	+++	+++	+++	+++	−	+++
SC117	+++	+++	+++	+++	+++	++	−	+++
SC142	+++	+++	+++	+++	+++	+++	−	+++
SC143	+++	+++	+++	+++	+++	++	−	+++
SC145	+++	+++	+++	+++	+++	++	−	+++
SC268	+++	+++	+++	+++	+++	+++	−	+++
SC417	+++	+++	+++	+++	+++	++	−	+++
SC523	+++	+++	+++	+++	+++	+++	−	+++
SC569	+++	+++	+++	+++	+++	+++	−	+++
SC600	+++	+++	+++	+++	+++	+++	−	+++
SC605	+++	+++	+++	+++	+++	+	−	+++
SC638	+++	+++	+++	+++	+++	++	−	+++
SC680	+++	+++	+++	+++	+++	+++	−	+++
*B. bifidum*								
JCM 1254	+++	+++	+++	+++	+++	−	++	+++
JCM 1255	−	+++	−	−	−	−	−	+++
JCM 1209	+++	+++	+++	+++	+++	−	++	−
JCM 7002	+++	+++	+++	+++	+++	+++	++	+++
JCM 7003	+++	+++	+++	+++	+++	+++	+++	+++
JCM 7004	+	++	++	++	++	++	++	++
S28-a	++	+++	+++	+++	+++	+++	−	+++
PRL2010	+++	+++	+++	+++	+++	+++	−	++
SC112	+++	+++	+++	+++	++	+++	+++	++
SC126	+++	+++	+++	++	++	++	++	++
SC555	+++	+++	+++	+++	+++	+++	++	++
SC572	+++	+++	+++	+++	+++	+++	++	+++
SC583	+++	+++	+++	++	++	++	+++	++
*B. animalis*								
JCM 10602	−	−	−	−	−	−	−	++

^a^Level of growth was classified as follows: −, negative (maximum OD_600_ < 0.250); +, low (OD_600_ from 0.250 to 0.500); ++, moderate (OD_600_ from 0.500 to 0.800); +++, high (OD_600_ > 0.800).

**Table 2:** Most highly transcribed genes in all datasets in *B. infantis.*

Feature ID	MeanRPKM	SDRPKM	Genelength	Description
NC_1013344_1014356	423108	175222	375	RNAse P
Blon_1922	11971	2505	1356	translation elongation factor Tu(EC:3.6.5.3)
Blon_2257	10261	5063	90	chaperonin Cpn10
Blon_0694	9911	5948	807	chaperonin GroEL
Blon_0679	7198	2063	1437	histone family protein DNA-binding protein
Blon_1722	6147	2759	1302	Fructose-6-phosphate phosphoketolase(EC:4.1.2.22)
Blon_0900	5758	2720	636	glyceraldehyde-3-phosphate dehydrogenase, type I(EC:1.2.1.12)
Blon_1095	5159	1997	4476	transcriptional regulator, Fis family(EC:2.2.1.2)
Blon_0308	4800	1373	1473	ATP synthase F1, gamma subunit(EC:3.6.3.14)
Blon_2152	4479	1208	747	phosphoglycerate mutase 1 family(EC:5.4.2.1)
Blon_0309	4369	1031	294	ATP synthase F1, beta subunit(EC:3.6.3.14)
Blon_1089	4310	978	1119	preprotein translocase, SecG subunit
Blon_2215	4041	1713	2181	translation initiation factor IF-1
Blon_0307	4036	975	924	ATP synthase F1, alpha subunit(EC:3.6.3.14)
Blon_2211	3882	972	912	DNA-directed RNA polymerase, alpha subunit(EC:2.7.7.6)
Blon_2475	3016	1753	450	ABC transporter related, ATPase component
Blon_0382	2871	1094	294	narrowly conserved hypothetical protein
Blon_1738	2421	1455	414	hypothetical protein

**Table 3:** Most highly transcribed genes in all datasets in *B. bifidum.*

Feature ID	MeanRPKM	SDRPKM	Genelength	Gene description
BBIF_01522	94,333	49,231	364	RNAse P
BBIF_00180	14,278	9,299	1308	ABC-type sugar transport system, periplasmic component
BBIF_01548	13,101	1,683	1200	translation elongation factor TU
BBIF_00999	8,704	7,171	282	Bacterial nucleoid DNA-binding protein
BBIF_01022	8,602	3,665	1791	ABC-type dipeptide transport system, periplasmic component
BBIF_00527	8,245	5,856	1551	PTS system, *N-*acetylglucosamine-specific IIBC component
BBIF_00151	7,302	3,843	1104	transaldolase, mycobacterial type(EC:2.2.1.2)
BBIF_01766	6,811	4,378	2376	formate acetyltransferase 1(EC:2.3.1.54)
BBIF_01046	6,234	1,175	1056	glyceraldehyde-3-phosphate dehydrogenase, type I(EC:1.2.1.12)
BBIF_01460	6,138	4,967	2757	Phosphoenolpyruvate carboxylase(EC:4.1.1.31)
BBIF_00286	5,645	3,686	2478	Phosphoketolase(EC:4.1.2.22)
BBIF_00528	5,155	3,411	507	PTS system, glucose subfamily, IIA component(EC:2.7.1.69)
BBIF_01456	4,752	2,330	564	peroxiredoxin(EC:1.11.1.15)
BBIF_01401	4,711	2,056	1128	ABC-type sugar transport systems, ATPase components
BBIF_00570	4,122	2,327	3339	*N-*acetyl-beta-hexosaminidase(EC:3.2.1.52)
BBIF_00150	3,780	1,866	2109	transketolase, bacterial and yeast(EC:2.2.1.1)
BBIF_00846	3,764	2,111	219	translation initiation factor IF-1
