# Erratum: Comparative transcriptomics reveals key differences in the response to milk oligosaccharides of infant gut-associated bifidobacteria

**DOI:** 10.1038/srep15311

**Published:** 2015-11-02

**Authors:** Daniel Garrido, Santiago Ruiz-Moyano, Danielle G. Lemay, David A. Sela, J. Bruce German, David A. Mills

Scientific Reports 5: Article number: 1351710.1038/srep13517; published online: 09042015; updated: 11022015

This Article contains typographical errors in Table 1.

For the *B. bifidum,* JCM1209 strain under ‘HMO’, ‘2FL’ and ‘3FL’, the level of growth should read ‘+’, ‘++’ and ‘+’ respectively.

In addition, for the *B. bifidum* PRL2010 strain under ‘6SL’ and ‘Mucin’, the level of growth should read ‘-’ and ‘++’ respectively.

