# Supplementary material for: Comparative transcriptomics reveals key differences in the response to milk oligosaccharides of infant gut-associated bifidobacteria
Source: Sci Rep. 2015 Sep 4;5:13517. doi: 10.1038/srep13517 (PMC4559671; doi:10.1038/srep13517)
Supplement: Supplementary Information [file srep13517-s1.pdf]

Comparative transcriptomics reveals key differences in the response to milk oligosaccharides of infant gut-associated bifidobacteria.

Daniel Garrido[1,3,5,6], Santiago Ruiz-Moyano[1,3], Danielle G. Lemay[3,4], David A. Sela[1,3,7], J. Bruce German[2,3], and David A. Mills[1,2,3] *.

[1]Department of Viticulture & Enology, [2]Food Science & Technology, [3]Foods for Health Institute and [4]Genome Center, University of California, One Shields Ave. Davis, CA 95616, United States; [5]Department of Chemical and Bioprocess Engineering, School of Engineering, and [6]Programa ASIS, Pontificia Universidad Católica de Chile, Av. Vicuña Mackenna 4860, Santiago, Chile; [7]Department of Food Science, University of Massachusetts, Amherst, MA 01003

SUPPLEMENTARY INFORMATION

Description: This file contains 19 Supplementary Tables and 2 Supplementary Figures.

TABLE S1: List of *Bifidobacterium* strains used in this study.

Code	Species	Additional strain information[a]	Source
JCM1254	*B. bifidum*	DSM20082	Intestine of adult
JCM1255	*B. bifidum*	ATCC29521; DSM20456	Infant feces
JCM1209	*B. bifidum*	ATCC11863; DSM20082	Infant feces
JCM7002	*B. bifidum*		Infant feces
JCM7003	*B. bifidum*		Infant feces
JCM7004	*B. bifidum*		Infant feces
S28-a	*B. bifidum*	Roy et al. 1996	Infant feces
PRL2010	*B. bifidum*	Turroni et al. 2010	Infant feces
SC112	*B. bifidum*	This study	Infant feces
SC126	*B. bifidum*	This study	Infant feces
SC555	*B. bifidum*	This study	Infant feces
SC572	*B. bifidum*	This study	Infant feces
SC583	*B. bifidum*	This study	Infant feces
ATCC15697	*B. longum subsp. infantis*	JCM1222; DSM20088	Infant feces
ATCC25962	*B. longum subsp. infantis*	JCM1210; DSM20223	Infant feces
ATCC17930	*B. longum subsp. infantis*	JCM1260; DSM20218	Infant feces
ATCC15702	*B. longum subsp. infantis*	JCM1272; DSM20090	Infant feces
JCM7007	*B. longum subsp. infantis*	LMG18901	Infant feces
JCM7009	*B. longum subsp. infantis*	LMG18902	Infant feces
JCM7011	*B. longum subsp. infantis*		Infant feces
SC30	*B. longum subsp. infantis*	This study	Infant feces
SC97	*B. longum subsp. infantis*	This study	Infant feces
SC117	*B. longum subsp. infantis*	This study	Infant feces
SC142	*B. longum subsp. infantis*	This study	Infant feces
SC143	*B. longum subsp. infantis*	This study	Infant feces
SC145	*B. longum subsp. infantis*	This study	Infant feces
SC268	*B. longum subsp. infantis*	This study	Infant feces
SC417	*B. longum subsp. infantis*	This study	Infant feces
SC523	*B. longum subsp. infantis*	This study	Infant feces
SC569	*B. longum subsp. infantis*	This study	Infant feces
SC600	*B. longum subsp. infantis*	This study	Infant feces
SC605	*B. longum subsp. infantis*	This study	Infant feces
SC638	*B. longum subsp. infantis*	This study	Infant feces
SC680	*B. longum subsp. infantis*	This study	Infant feces
JCM10602	*B. animalis subsp. lactis*	DSMZ 10140	Dairy product

[a]The original strain numbers are also noted, if known. JCM, Japan Collection of Microorganisms, ATCC, American Type Culture Collection; DSMZ, German Collection of Microorganisms and Cell Culture.

TABLE S2: MLST genes and primers.

Gene	PCR primer (5´-3´)*[,a]	Expected Amplicon size (bp)	Anneling Temp. (ºC)
clpC	GAG TAC CGC AAG TAC ATC GAG	748	63
	CAT CCT CAT CGT CGA ACA GGA AC		
purF	CAT TCG AAC TCC GAC ACC GA	977	62
	GTG GGG TAG TCG CCG TTG		
gyrB	AGC TGC ACG CBG GCG GCA AGT TCG	811	66
	GTT GCC GAG CTT GGT CTT GGT CTG		
fusA	ATC GGC ATC ATG GCY CAC ATY GAT	784	66
	CCA GCA TCG GCT GMA CRC CCT T		
Iles	ATC CCG CGY TAC CAG ACS ATG	789	66
	CGG TGT CGA CGT AGT CGG CG		
rplB	GGA CAA GGA CGG CRT SCC SGC CAA	498	67
	ACG ACC RCC GTG CGG GTG RTC GAC		
rpoB	GGC GAG CTG ATC CAG AAC CA	1057	62
	GCA TCC TCG TAG TTG TAS CC		

*= Upper sequence, forward primer; Lower sequence, reverse primer.

[a] In the primer sequence R indicates (A/G), S (C/G), Y (C/T).

TABLE S3: Descriptive evolutionary analysis of MLST data.

Gene	Fragment analyzed(bp)*	G+C (mol%)	polymorphic sites	Allele frequencies	π	k
			Bifidobacterium bifidum			
clpC	717 (0,26)	64,8	9	6	0,00356	2,54945
purF	875 (0,57)	64,4	16	12	0,00325	2,84615
gyrB	724 (0,35)	61,1	9	7	0,00354	2,5604
fusA	748(0,35)	62,9	14	10	0,0048	3,5934
Iles	743(0,22)	63,2	17	10	0,00618	4,5934
rplB	422 (0,50)	66,3	2	3	0,00138	0,58242
rpoB	984 (0,27)	65	18	11	0,00474	4,65934
			Bifidobacterium infantis			
clpC	721 (0,27)	62,55	30	8	0,01297	9,35238
purF	883 (0,58)	64,21	65	9	0,02431	21,467
gyrB	711 (0,34)	64,56	41	7	0,01932	13,733
fusA	766(0,36)	62,01	42	9	0,01862	14,26667
Iles	757(0,22)	62,88	55	8	0,02234	16,686
rplB	427 (0,51)	65,81	9	7	0,00567	2,419
rpoB	965 (0,27)	64,46	39	8	0,00754	7,276

*Percentage of the gene is given in parenthesis.

π = mean pairwise nucleotide difference per site.

k = mean pairwise nucleotide difference per sequence.

TABLE S4: Allelic profiles of 13 *B. bifidum* strains analyzed by MLST.

Strains	ST[a]	Allele						
		clpC	*purF*	*gyrB*	*fusA*	*Iles*	*rplB*	*rpoB*
JCM1254	1	1	2	4	1	10	2	9
S28-a	1	1	2	4	1	10	2	9
PRL2010	2	2	1	6	7	2	3	7
JCM1255	3	6	12	3	2	6	3	2
JCM1209	4	5	6	1	6	8	3	1
JCM7002	5	3	11	7	3	1	3	5
JCM7003	6	4	9	2	8	4	3	4
JCM7004	7	6	8	1	5	10	3	9
SC112	8	6	3	3	10	3	1	8
SC126	9	6	10	5	9	9	2	6
SC555	10	6	5	4	2	5	3	10
SC572	11	6	4	3	7	7	3	11
SC583	12	6	7	7	4	5	3	3

[a]ST Indicates specific sequence type.

TABLE S5: Allelic profiles of 15 *B. infantis* strains analyzed by MLST.

Strains	ST[a]	Allele						
		clpC	*purF*	*gyrB*	*fusA*	*Iles*	*rplB*	*rpoB*
ATCC15697	1	1	1	1	3	1	1	8
SC30	2	2	2	1	1	2	2	5
SC97	3	2	8	1	1	2	2	5
SC117	4	3	9	2	7	3	3	7
SC142	5	4	8	3	4	4	2	5
SC143	6	4	2	1	4	4	2	5
SC145	7	5	7	1	6	5	4	6
SC268	8	6	5	3	2	6	5	4
SC417	9	2	6	1	1	2	2	5
SC523	10	6	5	4	2	6	5	4
SC569	11	7	4	1	5	7	6	3
SC600	12	2	3	5	8	2	7	2
SC605	12	2	3	5	8	2	7	2
SC638	12	2	3	5	8	2	7	2
SC680	13	8	3	7	9	8	5	1

[a]ST Indicates specific sequence type.

Table S6: Genome statistics of the draft genome of *B. bifidum* SC555

	Number	% of Total
DNA, total number of bases	2237461	100.00%
DNA coding number of bases	1933914	86.43%
DNA G+C number of bases	1401692	62.65% [1]
DNA scaffolds	22	100.00%
Average lenght contigs	101703	
Genes total number	1894	100.00%
Protein coding genes	1835	96.88%
RNA genes	59	3.12%
rRNA genes[a]	3	0.16%
5S rRNA	1	0.05%
16S rRNA	1	0.05%
23S rRNA	1	0.05%
tRNA genes	53	2.80%
Other RNA genes	3	0.16%
Protein coding genes with function prediction	1363	71.96%
without function prediction	472	24.92%
Protein coding genes with enzymes	516	27.24%
w/o enzymes but with candidate KO based enzymes	29	1.53%
Protein coding genes connected to Transporter Classification	212	11.19%
Protein coding genes connected to KEGG pathways[3]	537	28.35%
not connected to KEGG pathways	1298	68.53%
Protein coding genes connected to KEGG Orthology (KO)	907	47.89%
not connected to KEGG Orthology (KO)	928	49.00%
Protein coding genes connected to MetaCyc pathways	502	26.50%
not connected to MetaCyc pathways	1333	70.38%
Protein coding genes with COGs[3]	1354	71.49%
with KOGs[3]	677	35.74%
with Pfam[3]	1447	76.40%
with TIGRfam[3]	593	31.31%
with IMG Terms	166	8.76%
with IMG Pathways	96	5.07%
with IMG Parts List	66	3.48%
in paralog clusters	427	22.54%

in Chromosomal Cassette	1894	100.00%
Chromosomal Cassettes	249	-
Biosynthetic Clusters	9	-
Genes in Biosynthetic Clusters	66	3.48%
Fused Protein coding genes	3	0.16%
Protein coding genes coding signal peptides	108	5.70%
Protein coding genes coding transmembrane proteins	459	24.23%
COG clusters	982	0.00%
KOG clusters	487	0.00%
Pfam clusters	1251	0.00%
TIGRfam clusters	574	0.00%

[a] Since SC555 is a draft genome, rRNA number is just a reference

Table S7: Gene number of putative HMO-genes in the genomes of *B. bifidum* SC555 and *B. bifidum* PRL2010.

	Solute Binding Protein Family 1	Sialidase (GH33)	α-Fucosidase (GH29)	α-Fucosidase (GH95)	β-Hexosaminidase (GH20)	Lacto-N-biosidase	β-Galactosidase GH2 (2334)	β-Galactosidase (GH42)
SC555	2	2	1	1	3	1	3	2
PRL2010	3	2	1	1	3	1	3	2

Table S8: General features of the RNA-seq experiments

Sample	Number of reads	Counted fragments to genes	Counted fragments to intergenic [a]	Uncounted fragments [b]	% uncounted	% reads aligning to genes	% Intergenic	%16S	%23S	%5S
B. infantis experiment										
LAC-A	9580821	6593523	1028625	1958673	20.44	68.82	10.74	0.38	2.57	0.01
LAC-B	8509364	5915002	895479	1698883	19.96	69.51	10.52	0.35	2.60	0.03
HMOearly-A	7431284	6008022	628754	794508	10.69	80.85	8.46	0.26	1.81	0.01
HMOearly-B	7869591	6394589	652910	822092	10.45	81.26	8.30	0.24	1.59	0.01
HMOmid-A	11927818	8563335	2084420	1280063	10.73	71.79	17.48	0.05	0.17	0.01
HMOmid-B	12697748	9421352	2021218	1255178	9.89	74.20	15.92	0.04	0.18	0.01
HMOlate-A	10085888	7409454	1580720	1095714	10.86	73.46	15.67	0.03	0.13	0.01
HMOlate-B	8538230	6526725	1190321	821184	9.62	76.44	13.94	0.10	0.28	0.02
LNT-A	10348666	8226643	1037151	1084872	10.48	79.49	10.02	2.12	2.54	0.00
LNT-B	9872022	8035279	935991	900752	9.12	81.39	9.48	1.73	2.06	0.00
LNnT-A	15999785	8613627	2890595	4495563	28.10	53.84	18.07	0.02	0.24	0.00
LNnT-B	15999800	7889369	2742892	5367539	33.55	49.31	17.14	0.03	0.43	0.01
2FL-A	11834663	8320115	2235223	1279325	10.81	70.30	18.89	0.01	0.15	0.00

2FL-B	7999912	5625018	1510477	864417	10.81	70.31	18.88	0.02	0.34	0.01
3FL-A	8249294	6065228	1423663	760403	9.22	73.52	17.26	0.02	0.32	0.02
3FL-B	12352540	9300426	1831905	1220209	9.88	75.29	14.83	0.01	0.20	0.01
6SL-A	9626876	6611304	1935766	1079806	11.22	68.68	20.11	0.02	0.47	0.03
6SL-B	8959521	5237849	2577162	1144510	12.77	58.46	28.76	0.04	1.00	0.16
B.bifidum experiment										
LAC-A	6832262	5063710	1155573	612979	8.97	74.11	16.91	0.02	0.38	0.01
LAC-B	9743954	7404364	1501297	838293	8.60	75.99	15.41	0.05	0.55	0.04
HMOearly-A	7137336	5796097	492958	848281	11.89	81.21	6.91	0.10	1.65	0.07
HMOearly-B	9440882	7717524	677384	1045974	11.08	81.75	7.18	0.06	1.12	0.03
HMOmid-A	5700702	4524342	533781	642579	11.27	79.36	9.36	0.02	1.10	0.03
HMOmid-B	5943078	4830773	525726	586579	9.87	81.28	8.85	0.14	1.35	0.02
HMOlate-A	8268172	6684808	807839	775525	9.38	80.85	9.77	0.09	0.92	0.02
HMOlate-B	6142644	4973114	637948	531582	8.65	80.96	10.39	0.07	1.57	0.03
LNT-A	7525773	4944007	1708234	873532	11.61	65.69	22.70	0.01	0.29	0.01
LNT-B	3199964	2113113	707284	379567	11.86	66.04	22.10	0.08	1.57	0.09
LNnT-A	3149953	2145609	680370	323974	10.29	68.12	21.60	0.06	0.95	0.01
LNnT-B	21902887	16216489	3132582	2553816	11.66	74.04	14.30	0.06	0.64	1.02

2FL-A	9647475	7141735	1687548	818192	8.48	74.03	17.49	0.02	0.40	0.02
2FL-B	9782588	7335764	1658551	788273	8.06	74.99	16.95	0.01	0.44	0.06
3FL-A	9562131	7163248	1511244	887639	9.28	74.91	15.80	0.04	0.67	0.04
3FL-B	9335729	6902333	1569093	864303	9.26	73.93	16.81	0.03	0.54	0.01
6SL-A	12108746	9491459	1487133	1130154	9.33	78.39	12.28	0.02	0.25	0.02
6SL-B	8108806	6356291	995662	756853	9.33	78.39	12.28	0.04	0.55	0.06
MUC-A	11392726	8978246	1257843	1156637	10.15	78.81	11.04	0.03	0.47	0.21
MUC-B	12561151	10048642	1489804	1022705	8.14	80.00	11.86	0.02	0.39	0.39
Summary		Reads aligning to genes	Reads aligning to IGR	Uncounted reads	% uncounted	% reads align genes	% Intergenic	%16S	%23S	%5S
Total reads B.infantis	187883823	130756860								
Average B. infantis	10437990	7264270	1622404	1551316	13.81	70.94	15.25	0.30	0.95	0.02
SD	2572943	1309715	708267	1277312	7.04	9.04	5.16	0.60	0.95	0.03
Total reads B. bifidum	177486959	135831668								
Average B. bifidum	8874348	6791583	1210893	871872	9.86	76.14	14.00	0.05	0.79	0.11

SD	4002486	3049299	633710	454424	1.29	4.96	4.80	0.03	0.45	0.23

[a] Total reads aligning to intergenic regions, identified in both genomes.

[b] Total reads not aligned to any genic or intergenic region.

Table S9: Function of genes associated to HMO consumption in *B. infantis* described in Figure 4

Locus tag	Gene lenght	Annotation	Possible function [a]
Blon_0344	978	Mandelate racemase/muconate lactonizing enzyme, N-terminal domain protein	Fucose metabolism
Blon_0247	1206	major facilitator superfamily MFS_1	HMO import
Blon_0248	1218	Alpha-L-fucosidase(EC:3.2.1.51)	Fucose metabolism
Blon_2177	696	extracellular solute-binding protein, family 1	HMO transport
Blon_2016	1407	Beta-galactosidase(EC:3.2.1.23)	HMO enzymatic degradation
Blon_0732	768	glycoside hydrolase, family 20	HMO enzymatic degradation
Blon_2332	1593	sugar (Glycoside-Pentoside-Hexuronide) transporter	HMO transport
Blon_2351	1437	extracellular solute-binding protein, family 1	ABC transporter for HMO
Blon_2350	2349	extracellular solute-binding protein, family 1	ABC transporter for HMO
Blon_2352	438	extracellular solute-binding protein, family 1	ABC transporter for HMO
Blon_2348	1356	Exo-alpha-sialidase(EC:3.2.1.18)	HMO enzymatic degradation
Blon_2349	3072	dihydrodipicolinate synthetase(EC:4.2.1.52)	Fucose metabolism
Blon_0538	75	UDP-glucose 4-epimerase(EC:5.1.3.2)	Galactose metabolism
Blon_2331	1317	sugar (Glycoside-Pentoside-Hexuronide) transporter	HMO transport
Blon_2354	792	extracellular solute-binding protein, family 1	HMO transport
Blon_2355	1278	glycoside hydrolase, family 20	HMO enzymatic degradation
Blon_0346	630	hypothetical protein	HMO enzymatic degradation
Blon_0341	2835	binding-protein-dependent transport systems inner membrane component	ABC transporter for HMO
Blon_2204	1164	binding-protein-dependent transport systems inner membrane component	ABC transporter for HMO
Blon_0345	1410	major facilitator superfamily MFS_1	HMO transport
Blon_2202	1539	extracellular solute-binding protein, family 1	HMO transport
Blon_0343	912	extracellular solute-binding protein, family 1	HMO transport
Blon_0342	519	binding-protein-dependent transport systems inner membrane component	HMO transport
Blon_2203	1125	binding-protein-dependent transport systems inner membrane component	HMO transport
Blon_2475	450	ABC transporter related	ATPase for ABC transporters
Blon_2063	1365	galactose-1-phosphate uridylyltransferase(EC:2.7.7.12)	Galactose metabolism

Blon_2171	2061	UDP-glucose 4-epimerase(EC:5.1.3.2)	GlcNAc metabolism
Blon_2184	1317	phosphoglucomutase, alpha-D-glucose phosphate-specific(EC:5.4.2.2)	Galactose metabolism
Blon_2062	261	galactokinase(EC:2.7.1.6)	Galactose metabolism
Blon_2334	933	glycoside hydrolase family 2, TIM barrel(EC:3.2.1.23)	HMO enzymatic degradation
Blon_0879	813	ROK family protein	GlcNAc metabolism
Blon_2341	801	protein of unknown function DUF624	HMO transport
Blon_2353	897	hypothetical protein	HMO transport
Blon_2176	1284	binding-protein-dependent transport systems inner membrane component	HMO transport
Blon_2175	1704	binding-protein-dependent transport systems inner membrane component	HMO transport
Blon_2174	690	hypothetical protein	Galactose metabolism
Blon_2172	633	UDP-glucose--hexose-1-phosphate uridylyltransferase(EC:2.7.7.12,EC:2.7.7.10)	Galactose metabolism
Blon_2173	1602	aminoglycoside phosphotransferase(EC:2.7.1.162)	Galactose metabolism
Blon_0645	945	N-acylglucosamine-6-phosphate 2-epimerase(EC:5.1.3.9)	NeuAc metabolism
Blon_0644	912	ROK family protein(EC:2.7.1.2)	NeuAc metabolism
Blon_0646	684	glycosyl hydrolase, BNR repeat-containing protein(EC:3.2.1.18)	HMO enzymatic degradation
Blon_0881	1338	glucosamine-6-phosphate isomerase(EC:3.5.99.6)	GlcNAc metabolism
Blon_0882	912	N-acetylglucosamine-6-phosphate deacetylase(EC:3.5.1.25)	GlcNAc metabolism
Blon_2347	1533	extracellular solute-binding protein, family 1	HMO transport
Blon_2344	1200	extracellular solute-binding protein, family 1	HMO transport
Blon_2342	1296	binding-protein-dependent transport systems inner membrane component	HMO transport
Blon_2345	675	binding-protein-dependent transport systems inner membrane component	HMO transport
Blon_2343	2238	binding-protein-dependent transport systems inner membrane component	HMO transport
Blon_2346	1524	binding-protein-dependent transport systems inner membrane component	HMO transport
Blon_2337	1347	RbsD or FucU transport(EC:5.1.3.-)	Fucose metabolism
Blon_2338	1116	dihydrodipicolinate synthetase(EC:4.2.1.52)	Fucose metabolism
Blon_2335	1254	alpha-1/2-fucosidase	HMO enzymatic degradation
Blon_2336	1326	alpha-1,3/4-fucosidase, putative(EC:3.2.1.51)	HMO enzymatic degradation
Blon_2339	690	short-chain dehydrogenase/reductase SDR(EC:1.1.1.100)	Fucose metabolism

Blon_2340	1404	Mandelate racemase/muconate lactonizing enzyme, C-terminal domain protein(EC:5.1.2.2)	Fucose metabolism

[a] Possible function was proposed from previous works referenced throughout the manuscript, as well as gene annotation.

Table S10: Function of genes associated to HMO consumption in *B. bifidum* described in Figure 5.

Locus tag	Gene lenght	Annotation	Possible function
BBIF_01022	1.791	ABC-type dipeptide transport system, periplasmic component	HMO transport
BBIF_00180	1.308	ABC-type sugar transport system, periplasmic component	HMO transport
BBIF_00172	1.023	UDP-glucose-4-epimerase(EC:5.1.3.2)	Galactose metabolism
BBIF_00503	5.796	PEGA domain./Uncharacterised Sugar-binding Domain./F5/8 type C domain.	HMO enzymatic degradation
BBIF_01401	1.128	ABC-type sugar transport systems, ATPase components	ATPase for ABC transporters
BBIF_00527	1.551	PTS system, N-acetylglucosamine-specific IIBC component	HMO transport
BBIF_00528	507	PTS system, glucose subfamily, IIA component(EC:2.7.1.69)	HMO transport
BBIF_01068	813	glucosamine-6-phosphate isomerase(EC:3.5.99.6)	GlcNAc metabolism
BBIF_00546	3159	Beta-galactosidase/beta-glucuronidase(EC:3.2.1.23)	HMO enzymatic degradation
BBIF_00547	1476	sugar (Glycoside-Pentoside-Hexuronide) transporter	HMO transport
BBIF_01155	5.562	Beta-galactosidase/beta-glucuronidase(EC:3.2.1.23)	HMO enzymatic degradation
BBIF_01261	5.880	Bacterial surface proteins containing Ig-like domains	HMO enzymatic degradation
BBIF_00173	1.515	Galactose-1-phosphate uridyltransferase(EC:2.7.7.12)	Galactose metabolism
BBIF_00174	1.089	Putative homoserine kinase type II (protein kinase fold)	Galactose metabolism
BBIF_01067	1.257	N-acetylglucosamine-6-phosphate deacetylase(EC:3.5.1.25)	GlcNAc metabolism
BBIF_00178	951	ABC-type sugar transport system, permease component	HMO transport
BBIF_00179	972	ABC-type sugar transport systems, permease components	HMO transport
BBIF_00177	2.256	1,3-beta-galactosyl-N-acetylhexosamine phosphorylase	HMO enzymatic degradation
BBIF_00369	1251	galactose-1-phosphate uridylyltransferase, family 1(EC:2.7.7.12)	GlcNAc metabolism
BBIF_01218	456	Lacto-N-biose phosphorylase.	HMO enzymatic degradation
BBIF_00871	1677	phosphoglucomutase, alpha-D-glucose phosphate-specific(EC:5.4.2.2)	Galactose metabolism
BBIF_01021	1.431	Aminopeptidase C(EC:3.4.22.40)	Galactose metabolism
BBIF_01217	333	Lacto-N-biose phosphorylase.	HMO enzymatic degradation
BBIF_00368	1.251	galactokinase(EC:2.7.1.6)	Galactose metabolism

BBIF_00533	1.155	Lacto-N-biose phosphorylase.	HMO enzymatic degradation
BBIF_01442	3498	Alpha-L-arabinofuranosidase	HMO enzymatic degradation
BBIF_00707	2505	NPCBM-associated, NEW3 domain of alpha-galactosidase./BNR/Asp-box repeat.(EC:3.2.1.18)	HMO enzymatic degradation
BBIF_00008	4.500	Alpha-L-fucosidase(EC:3.2.1.51)	HMO enzymatic degradation
BBIF_01050	957	Galactose mutarotase and related enzymes	Galactose metabolism
BBIF_00852	4884	N-acetyl-beta-hexosaminidase(EC:3.2.1.52)	HMO enzymatic degradation
BBIF_00176	942	Transcriptional regulator/sugar kinase(EC:2.7.1.2)	GlcNAc metabolism
BBIF_00136	3183	N-acetyl-beta-hexosaminidase	HMO enzymatic degradation
BBIF_00175	1155	Transcriptional regulator/sugar kinase	GlcNAc metabolism
BBIF_01445	2337	N-acetyl-beta-hexosaminidase	HMO enzymatic degradation
BBIF_00550	1014	UDP-glucose-4-epimerase(EC:5.1.3.2)	Galactose metabolism
BBIF_01073	1284	Fucose permease	HMO transport
BBIF_00706	5388	GDSL-like Lipase/Acylhydrolase./Laminin G domain./BNR/Asp-box repeat.(EC:3.2.1.18)	HMO enzymatic degradation

[a] Possible function was proposed from previous works referenced throughout the manuscript, as well as gene annotation.

Table S11: Enriched annotations, *B. infantis* genes UP-regulated in relative to lactose

Type	ANNOT_ID	ANNOT_DESC	HMO-E	HMO-M	HMO-L	LNT	LNnT	2FL	3FL	6SL
COG	COG1175	ABC-type sugar transport systems, permease components	NS	NS	NS	NS	0.059	0.019	NS	0.09
COG	COG0395	ABC-type sugar transport system, permease component	0.041	NS	NS	0.027	NS	0.08	0.015	0.024
COG	COG2801	Transposase and inactivated derivatives	NS	NS	NS	NS	NS	NS	NS	0.029
COG	COG2197	Response regulator containing a CheY-like receiver domain and an HTH DNA-binding domain	NS	NS	NS	0.046	0.04	NS	NS	NS
COG	COG1653	ABC-type sugar transport system, periplasmic component	0.004	NS	0.006	0.063	0.08	0.003	0.062	0
GO	GO:0005215	transporter activity	0	NS	NS	NS	0.011	NS	NS	0.007
GO	GO:0005975	carbohydrate metabolic process	NS	NS	0.008	NS	NS	NS	NS	NS
GO	GO:0043565	sequence-specific DNA binding	NS	NS	NS	0	NS	NS	NS	NS
GO	GO:0008643	carbohydrate transport	0.003	NS	NS	0.01	0.01	0.001	0.001	0
SRI	TRNA CHARGING		NS	0.005	NS	NS	NS	NS	NS	NS

Table S12: Enriched annotations, *B. infantis* genes DOWN-regulated in relative to lactose

Type	ANNOT_ID	ANNOT_DESC	HMO-E	HMO-M	HMO-L	LNT	LNnT	2FL	3FL	6SL
GO	GO:0003723	RNA binding	NS	NS	NS	NS	NS	NS	0.017	NS
KEGG	MAP00240	Pyrimidine metabolism	NS	0.005	0.016	0.038	NS	NS	NS	NS
KEGG	MAP00230	Purine metabolism	NS	0.001	0.009	0.005	NS	NS	NS	NS
SRI	PEPTIDOGLYCAN BIOSYNTHESIS III MYCOBACTERIA		NS	NS	NS	NS	NS	NS	0.041	NS
SRI	TRNA CHARGING		NS	NS	NS	NS	NS	0.045	NS	NS

Table S13: Enriched annotations, *B. bifidum* genes UP-regulated relative to LACTOSE

Type	ANNOT_ID	ANNOT_DESC	HMO-E	HMO-M	HMO-L	MUC	LNT	LNnT	2'FL	3FL	6'SL
COG	COG1609	Transcriptional regulators	NS	NS	NS	NS	NS	0.041	NS	0.029	NS
GO	GO:0007155	cell adhesion	0.002	0.001	0.052	NS	NS	0	NS	NS	NS
GO	GO:0005975	carbohydrate metabolic process	0.006	0.05	NS	0.095	NS	0.087	NS	NS	NS
GO	GO:0008643	carbohydrate transport	NS	0.089	0.015	0.416	NS	0.126	NS	NS	NS
GO	GO:0019843	rRNA binding	0	NS	NS	0.102	NS	NS	NS	NS	NS
GO	GO:0006412	translation	0	0.158	NS	0.029	NS	NS	NS	NS	NS
GO	GO:0003735	structural constituent of ribosome	0	0.032	NS	0.004	NS	NS	NS	NS	NS
GO	GO:0005840	ribosome	0	0.163	NS	0.008	NS	NS	NS	NS	NS
GO	GO:0005351	sugar:hydrogen symporter activity	NS	NS	NS	0.042	NS	NS	NS	NS	NS
GO	GO:0009401	phosphoenolpyruvate-dependent sugar phosphotransferase system	NS	0.246	NS	0.027	NS	0.162	NS	NS	NS
GO	GO:0043169	cation binding	0.007	0.338	NS	NS	NS	0.042	NS	NS	NS
KEGG	MAP00520	Amino sugar and nucleotide sugar metabolism	NS	0.094	NS	0.022	NS	NS	NS	NS	NS
KEGG	MAP00330	Arginine and proline metabolism	NS	0.197	0.035	NS	NS	NS	NS	NS	NS
KEGG	MAP00250	Alanine, aspartate and glutamate metabolism	NS	0.043	0.033	NS	NS	NS	NS	NS	NS
KEGG	MAP00260	Glycine, serine and threonine metabolism	NS	NS	NS	NS	NS	NS	NS	NS	0.013
KEGG	MAP03010	Ribosome	0	0.01	0.079	0.001	NS	NS	NS	NS	NS
KEGG	MAP00511	Other glycan degradation	NS	0.01	NS	NS	NS	NS	NS	NS	NS
SRI	PWY-5686	uridine-5-phosphate biosynthesis	NS	0.045	0.013	NS	NS	NS	NS	NS	NS
SRI	TRNA-CHARGING-PWY	tRNA charging	NS	NS	NS	NS	0	0	NS	NS	NS
SRI	PWY0-162	pyrimidine ribonucleotides *de novo* biosynthesis	NS	0.081	0.049	NS	NS	NS	NS	NS	NS

Table S14: Enriched annotations, *B. bifidum* genes DOWN-regulated relative to LACTOSE

Type	ANNOT_ID	ANNOT_DESC	HMO-E	HMO-M	HMO-L	MUC	LNT	LNnT	2'FL	3FL	6'SL
GO	GO:0006281	DNA repair	NS	NS	NS	NS	NS	0.016	NS	NS	NS
KEGG	MAP03010	Ribosome	NS	NS	NS	NS	NS	NS	0.008	NS	NS
KEGG	MAP00190	Oxidative phosphorylation	NS	NS	NS	0.012	NS	NS	NS	NS	NS
SRI	TRNA-CHARGING-PWY	tRNA charging	NS	NS	NS	NS	NS	NS	0	0.028	NS

Table S15: Number of orthologs differentially regulated between *B. bifidum* and *B. infantis* in response to the same substrate

Substrate	# DE orthologs	# Up in Infantis	# Up in Bifidum
LAC	1003	509	494
HMO early	988	504	484
HMO mid	902	448	454
HMO late	995	491	504
LNT	1113	554	559
LNnT	863	434	429
2'FL	1078	550	528
3'FL	1034	520	514
6'SL	772	387	385

* DE: Differentially expressed

Table 16: Orthologs highly up-regulated in *B. bifidum* relative to *B. infantis* in response to HMO (early time point)

Mean Counts B. infantis	MeanCounts B. bifidum	Fold Change	Adjusted p-value	Infantis Ortholog	Bifidum Ortholog	Product Name
1218.4	180768.6	148.4	0	Blon_2471	BBIF_00527	PTS system, *N*-acetylglucosamine-specific IIBC subunit
214.7	29899.2	139.3	0	Blon_2470	BBIF_00528	PTS system, glucose subfamily, IIA subunit
379.6	35529.9	93.6	0	Blon_2176	BBIF_00179	binding-protein-dependent transport systems inner membrane component
2191.6	183539.1	83.7	0	Blon_2177	BBIF_00180	extracellular solute-binding protein, family 1
395.4	32137.9	81.3	0	Blon_2175	BBIF_00178	binding-protein-dependent transport systems inner membrane component
747.3	44345.8	59.3	0	Blon_2174	BBIF_00177	hypothetical protein
794.6	22423.6	28.2	5.30E-166	Blon_1694	BBIF_01584	protein of unknown function DUF214
52.1	1227.7	23.6	1.05E-172	Blon_1605	BBIF_01817	Phosphoglycerate mutase
251.1	5653.3	22.5	0	Blon_1695	BBIF_01583	ABC transporter related
88.8	1941.7	21.9	1.08E-234	Blon_2046	BBIF_00354	hypothetical protein
471.2	10236.9	21.7	0	Blon_2173	BBIF_00174	aminoglycoside phosphotransferase
253.7	5132.2	20.2	2.96E-25	Blon_0197	BBIF_00642	narrowly conserved hypothetical protein
537.1	10706.1	19.9	0	Blon_2172	BBIF_00173	UDP-glucose--hexose-1-phosphate uridylyltransferase
53.9	1004.8	18.7	4.91E-136	Blon_2042	BBIF_01215	ATPase associated with various cellular activities, AAA_3
179.4	3293.2	18.4	1.45E-142	Blon_0177	BBIF_00649	Phosphotransferase system,

Mean Counts<br><br>B. infantis	MeanCounts<br><br>B. bifidum	Fold<br><br>Change	Adjusted p-value	Infantis<br><br>Ortholog	Bifidum<br><br>Ortholog	Product<br><br>Name
						phosphocarrier protein HPr
1098.1	15143.5	13.8	0	Blon_2322	BBIF_00631	FAD-dependent pyridine nucleotide-disulphide oxidoreductase
92.5	1268.8	13.7	2.12E-144	Blon_2044	BBIF_00352	transglutaminase domain protein
36.4	470.5	12.9	3.02E-63	Blon_2043	BBIF_00351	protein of unknown function DUF58
166.8	2112.5	12.7	3.78E-197	Blon_0509	BBIF_00169	integral membrane sensor signal transduction histidine kinase
189.1	2310.3	12.2	1.06E-19	Blon_2411	BBIF_01430	glycoside hydrolase, family 43
148.9	1738.3	11.7	1.52E-165	Blon_2047	BBIF_00355	FHA domain containing protein
144.3	1572.1	10.9	7.57E-71	Blon_2003	BBIF_01861	Cystathionine gamma-synthase
70.9	769.5	10.9	9.66E-88	Blon_1376	BBIF_00234	VanZ family protein

Table 17: Orthologs highly up-regulated in *B. infantis* relative to *B. bifidum* in response to HMO (early time point)

Mean Counts Infantis	Mean Counts Bifidum	Fold Change	Adjusted p-value	Infantis Ortholog	Bifidum Ortholog	Product Name
10420.8	79.6	130.9	0	Blon_1721	BBIF_01591	NLPA lipoprotein
16909.8	151.1	111.9	0	Blon_2339<br>Blon_2308	BBIF_01220	short-chain dehydrogenase/reductase SDR
3830.4	76.0	50.4	0	Blon_1720	BBIF_01594	ABC transporter related
8573.4	178.5	48	0	Blon_1905	BBIF_01559	Beta-glucosidase
1924.1	46.2	41.6	4.43E-264	Blon_0342<br>Blon_2203	BBIF_00011	binding-protein-dependent transport systems inner membrane component
1920.5	62.9	30.5	2.39E-246	Blon_1719	BBIF_01595	binding-protein-dependent transport systems inner membrane component
529.2	32.0	16.5	1.81E-72	Blon_1053	BBIF_00193	hypothetical protein
1194.0	90.5	13.2	1.77E-130	Blon_0449<br>Blon_1356<br>Blon_2279	BBIF_01373	Integrase, catalytic region
6951.8	543.5	12.8	0	Blon_1232<br>Blon_1233	BBIF_01490<br>BBIF_00727	hypothetical protein
1011.5	93.1	10.9	1.77E-103	Blon_0821	BBIF_00404	carbohydrate kinase, FGGY
246.8	24.0	10.3	1.27E-31	Blon_0300	BBIF_01716	hypothetical protein
7981.0	785.2	10.2	1.66E-315	Blon_1454	BBIF_00063	5,10-methylenetetrahydrofolate reductase
208.5	20.7	10.1	1.77E-25	Blon_1423	BBIF_00221	amidohydrolase-like protein

Table S18: Number of differentially regulated genes in *B. bifidum* relative to lactose

Treatment	Number DE genes
2'FL	230
3'FL	138
6'FL	826
MUC	1129
LNT	1273
LNnT	1129
HMO Early	1170
HMO Mid	1280
HMO Late	1271

Table S19: Number of differentially regulated genes in *B. infantis* relative to lactose

Treatment	Number DE genes
2'FL	1148
3'FL	1277
6'FL	1385
LNT	992
LNnT	963
HMO Early	815
HMO Mid	605
HMO Late	1014

LEGEND TO SUPPLEMENTARY FIGURES

FIGURE S1: Evolutionary relationship of *B. infantis* (upper branch) and *B. bifidum* (lower branch) strains used in the study. The tree is drawn to scale, with branch lengths in the same units (number of base substitutions per site) as those of the evolutionary distances used to infer the phylogenetic tree. The evolutionary history was inferred using the Minimum Evolution method, followed by 1000 bootstrap replicates.

FIGURE S2: Venn diagram indicating the number of ortholog and unique genes in B. bifidum strains SC555 and PRL2010.

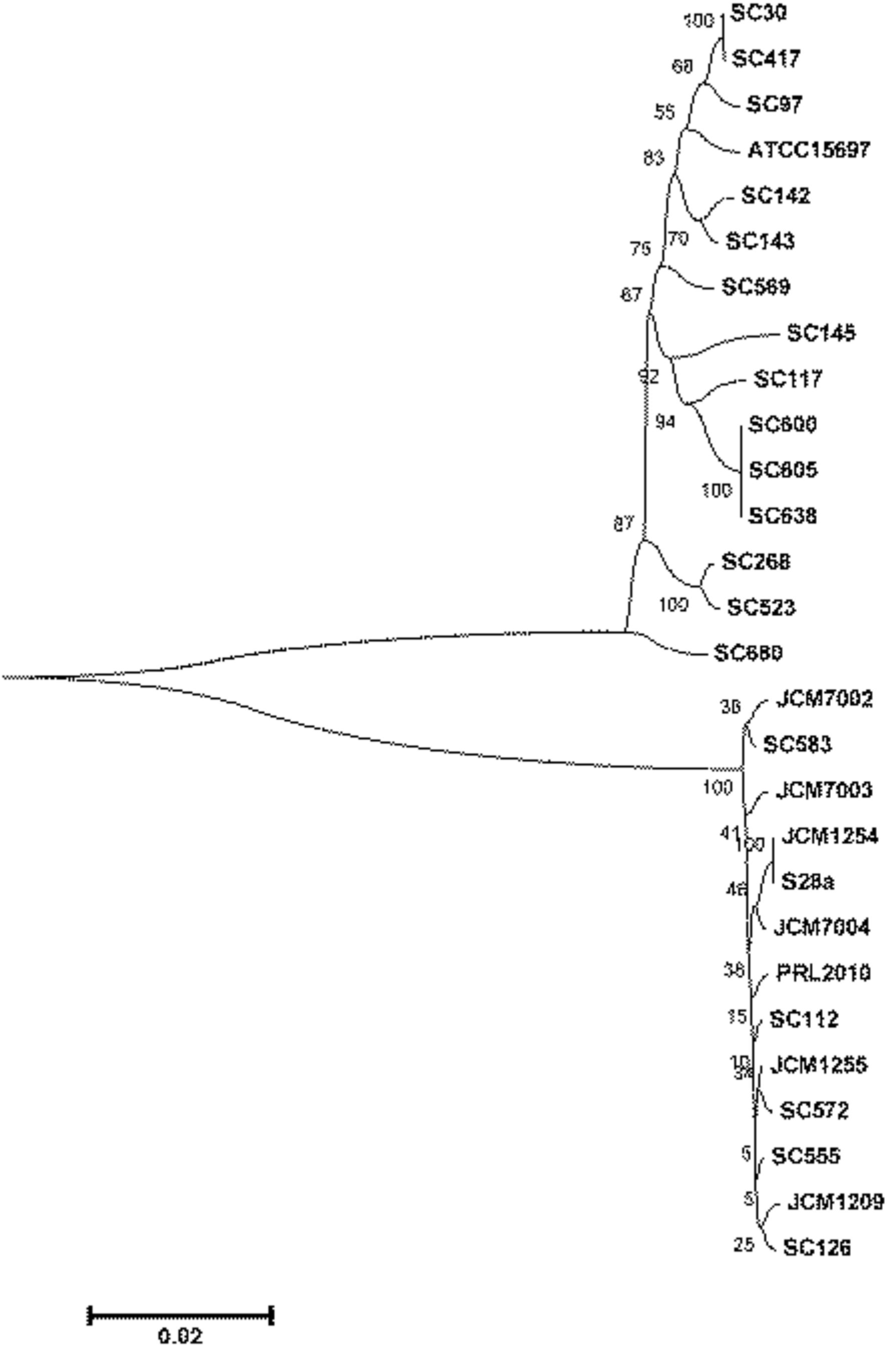
SC30
SC417
SC97
ATCC15697
SC142
SC143
SC569
SC145
SC117
SC600
SC605
SC638
SC268
SC523
SC680
JCM7002
SC583
JCM7003
JCM1254
S28a
JCM7004
PRL2010
SC112
JCM1255
SC572
SC555
JCM1209
SC126
0.02

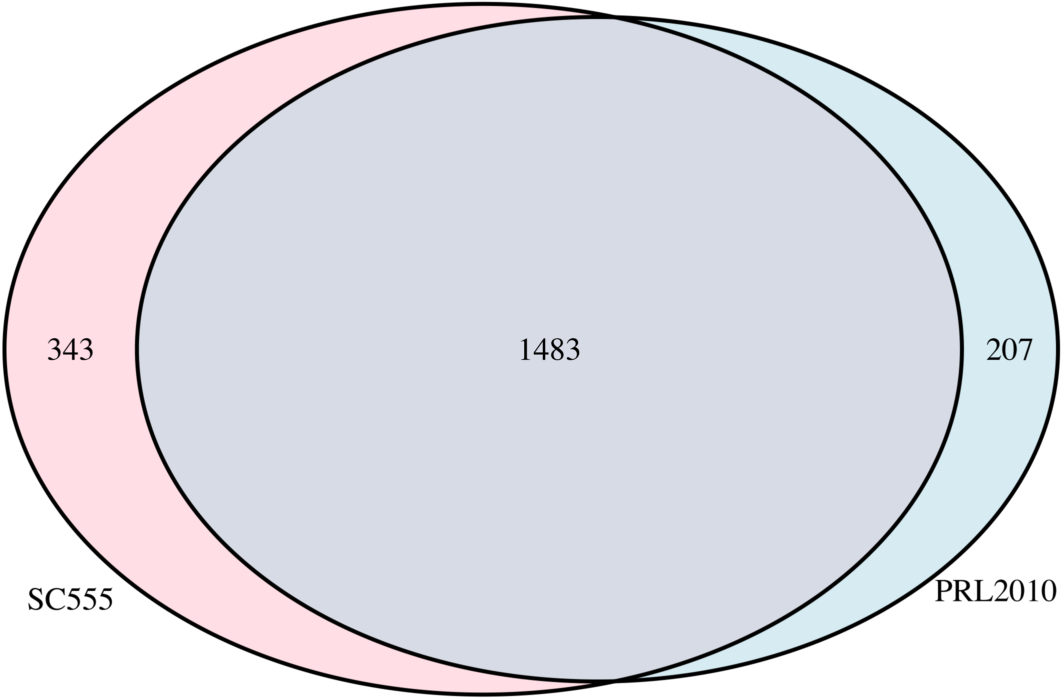
343
1483
207
SC555
PRL2010
